# Quantitative Analysis of Immune Response and Erythropoiesis during Rodent Malarial Infection

**DOI:** 10.1371/journal.pcbi.1000946

**Published:** 2010-09-30

**Authors:** Martin R. Miller, Lars Råberg, Andrew F. Read, Nicholas J. Savill

**Affiliations:** 1 Centre for Infectious Diseases, Institute of Immunology and Infection Research, University of Edinburgh, Edinburgh, United Kingdom; 2 Department of Animal Ecology, Lund University, Lund, Sweden; 3 Center for Infectious Disease Dynamics and Departments of Biology and Entomology, Pennsylvania State University, University Park, Pennsylvania, United States of America; University of New South Wales, Australia

## Abstract

Malarial infection is associated with complex immune and erythropoietic responses in the host. A quantitative understanding of these processes is essential to help inform malaria therapy and for the design of effective vaccines. In this study, we use a statistical model-fitting approach to investigate the immune and erythropoietic responses in *Plasmodium chabaudi* infections of mice. Three mouse phenotypes (wildtype, T-cell-deficient nude mice, and nude mice reconstituted with T-cells taken from wildtype mice) were infected with one of two parasite clones (AS or AJ). Under a Bayesian framework, we use an adaptive population-based Markov chain Monte Carlo method and fit a set of dynamical models to observed data on parasite and red blood cell (RBC) densities. Model fits are compared using Bayes' factors and parameter estimates obtained. We consider three independent immune mechanisms: clearance of parasitised RBCs (pRBC), clearance of unparasitised RBCs (uRBC), and clearance of parasites that burst from RBCs (merozoites). Our results suggest that the immune response of wildtype mice is associated with less destruction of uRBCs, compared to the immune response of nude mice. There is a greater degree of synchronisation between pRBC and uRBC clearance than between either mechanism and merozoite clearance. In all three mouse phenotypes, control of the peak of parasite density is associated with pRBC clearance. In wildtype mice and AS-infected nude mice, control of the peak is also associated with uRBC clearance. Our results suggest that uRBC clearance, rather than RBC infection, is the major determinant of RBC dynamics from approximately day 12 post-innoculation. During the first 2–3 weeks of blood-stage infection, immune-mediated clearance of pRBCs and uRBCs appears to have a much stronger effect than immune-mediated merozoite clearance. Upregulation of erythropoiesis is dependent on mouse phenotype and is greater in wildtype and reconstitited mice. Our study highlights the informative power of statistically rigorous model-fitting techniques in elucidating biological systems.

## Author Summary

Malaria is a disease caused by a protozoan parasite of the genus *Plasmodium*. Every year there are around 250 million human cases of malaria, resulting in around a million deaths. Most of the severe cases and deaths are due to *Plasmodium falciparum*, which is endemic in much of sub-Saharan Africa and other tropical areas. The pathology of malaria is related to the asexual stage of the parasite. Understanding the infection dynamics during this stage is therefore essential to inform malaria treatment and vaccine design. Experimental infections of rodents represent an important first step towards understanding the more complicated human infections. We developed a series of models representing different hypotheses about the main processes regulating the infection dynamics during the asexual stage. Models were fit to data on *Plasmodium chabaudi* infections of mice, using a Bayesian statistical framework. The accuracy of different models in explaining the RBC and parasite densities was quantified. We identify the role of different types of immune-mediated mechanism, and show that RBC production (erythropoiesis) increases during infection. Differences between mouse phenotypes are explained. Our study highlights the informative power of model-fitting techniques in explaining biological systems.

## Introduction

Malarial infection of humans is a major cause of morbidity and mortality, continuing to cause around 250 million cases and close to a million deaths annually [1]. The vast majority of severe cases and deaths are due to *Plasmodium falciparum*, which is endemic in most of sub-Saharan Africa and other tropical areas [2]. Although there is no simple relationship between the pathogenic processes and clinical syndromes, disease only begins once the asexual parasite begins to multiply within the host's red blood cells (RBCs) [3]. The asexual dynamics depend on a complex interaction between the malaria parasite and the host's immune and erythropoetic responses [4]. Experimental methods have helped elucidate key aspects of this interaction. Such factors include the parasite's destruction of RBCs due to reproduction [5], [6], immune-mediated clearance of merozoites and parasitised RBCs (pRBC) [7], [8], and immune-mediated clearance of unparasitised RBCs (uRBC). In particular, there is evidence that loss of uRBCs is responsible for the vast majority of the anaemia [9]–[11]. Suppression of RBC production (dyserythropoiesis) during the acute phase may also contribute to anaemia [12], [13], although recent modelling suggests that, overall, the level of erythropoiesis increases during malaria infection [8], [9], [14].

A full understanding of the infection dynamics requires quantitative analysis of the relative importance of the contributory factors [3]. Such an assessment is vital to help inform malaria treatment and intervention programmes [8], [15], [16]. In particular, the design of effective vaccines and immunotherapies depends largely on our understanding of the innate and adaptive immune responses [2], [17]. In this context, rodent malaria models allow a highly replicable, highly controlled experiment. Although there are important differences between rodent and human malarias, a quantitative understanding of the rodent system, where we can control both host and parasite genetics, should help our understanding of the human case in which controlled experiments are unethical.

As in other areas of science, mathematical models can be used to make inferences about complex dynamical systems by fitting them to data. This approach allows us to formally and quantitatively test and compare competing hypotheses, and to make quantitative predictions for future empirical testing. It is the most powerful and rapid way of culling possible, but incorrect, hypotheses. In the mathematical modelling literature on malaria, there are a number of studies that quantitatively fit models to data [6], [8], [9], [14], [15], [18]–[22]. However, the poorly developed statistical, diagnostic and computational methodologies of fitting nonlinear dynamical models to noisy data (see [23] and Discussion) meant that these studies had to focus on particular aspects of the host-pathogen system in isolation. The method used was maximum likelihood. Its application to nonlinear systems is problematic because the nonlinearities create complex multi-dimensional likelihood surfaces. Search algorithms easily become trapped in local maxima, leading to false inferences [24]. Even if one is reasonably sure of having found the global maximum, evaluating parameter confidence intervals and covariances is computationally expensive and laborious, and computing predictive intervals practically impossible [25].

Recent developments in adaptive, population-based Markov chain Monte Carlo (McMC) methods overcome all of the problems associated with maximum likelihood [24], [26]–[30]. The use of a Bayesian framework enables us to incorporate prior knowledge and uncertainty about the parameters. It allows us to quantify our relative belief in one model predicting the data over another, rather than accepting and rejecting models using conventional, but arbitrary, cut-offs. In order to use Bayesian statistics, we need to know the structure and variance of the measurement errors. Fortunately, these are known for our data sets.

In this study we develop a set of models to test competing hypotheses describing the asexual stage of the malaria parasite. We fit the models to a set of data on *Plasmodium chabaudi* infections [31] using an adaptive McMC algorithm. We provide parameter estimates, examine differences between mouse and parasite strains, and make quantitative predictions about the immune and erythropoietic systems' dynamics, and their effects on the RBC population. In modelling the asexual dynamics, there are three general processes we need to consider: (i) the infection of RBCs, (ii) the immune response, and (iii) the response of the erythropoietic system to malaria-induced anaemia.

The immune system's response to malaria is exceedingly complex and there is still much to learn about it qualitatively, let alone quantitatively [17]. Mathematical models have generally represented the immune response either as a single variable functionally linked to parasite density, or as separate innate and adaptive components [8], [21], [32]–[35]. The model of Recker et al. (2004) further discriminates, on the basis of human serologic data, between short-term, partially cross-reactive immune responses and long-term specific responses [36]. These models have given valuable insights into the immune dynamics, but it is important to acknowledge that the immune response consists of multiple arms, each targeting different aspects of the parasite [2]. Here we model the immune system as time-dependent immune-mediated clearance rates of merozoites, pRBCs and uRBCs. This allows us to bypass the debate about the highly interdependent innate and adaptive arms of the immune response, i.e., when they are activated, what they target, and how they develop over time, and instead focus on the functional consequences in terms of the infection dynamics.

We also draw attention to a key aspect of malaria asexual reproduction universally ignored in previous modelling studies. It is established that individual RBCs may be parasitised by more than one merozoite. Multiply-parasitised RBCs are often observed in experiments, but it is not known whether their subsequent behaviour is the same as that of singly-parasitised RBCs; previous models have generally assumed that their dynamics are identical. Here we test that assumption. In particular, we test whether multiply-parasitised RBCs have a greater death rate than other RBCs, and whether they produce a greater number of merozoites than singly-parasitised cells.

## Materials and Methods

### Previous experimental data

We used data obtained from a previous experiment [31]. Briefly, three different mice phenotypes were infected with either of two genetically distinct clones of *Plasmodium chabaudi* (AS or AJ). Both clones were originally isolated from thicket rats (*Thamnomys rutilans*) in the Central African Republic [37]. The AS clone is associated with a lower peak density relative to AJ; it also has lower virulence, as measured by anaemia and weight loss [38]. Three different phenotypes of BALB/c mice were used: (i) wildtype mice; (ii) *nu*/*nu* mice (“nude mice”; Harlan UK); and (iii) nude mice reconstituted with T cells taken from wildtype mice. The mutation *nu* is a recessive mutation that blocks the development of the thymus. Nude mice therefore lack mature T cells, whereas heterozygotes (*nu*/+) have a normal immune system [39]. Nude and reconstituted mice are smaller than the wildtype and are hairless.

Mice of each phenotype (wildtype, nude, reconstituted) were innoculated with 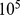 pRBCs of either AS or AJ. This gave six treatment groups. There were seven mice in both treatment groups for nude mice, and six mice in each treatment group for reconstituted and wildtype mice. Measurements of RBC and parasite density were taken on days 0, 2, 4, and then daily until day 18 when the experiment was terminated. Parasite density was measured daily at 08:00 hrs using quantitative PCR, at which point the asexual merozoites have yet to replicate within pRBCs. Both RBC and parasite densities are expressed in terms of the number per microlitre (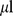) of blood. Full details of the experimental methods are given in [31]. We removed a single data point from one of the reconstituted AJ-infected replicates. This mouse had much lower parasite density on day 14 than all the other mice. The data point was therefore considered to be an outlier.

**Figure 1:**
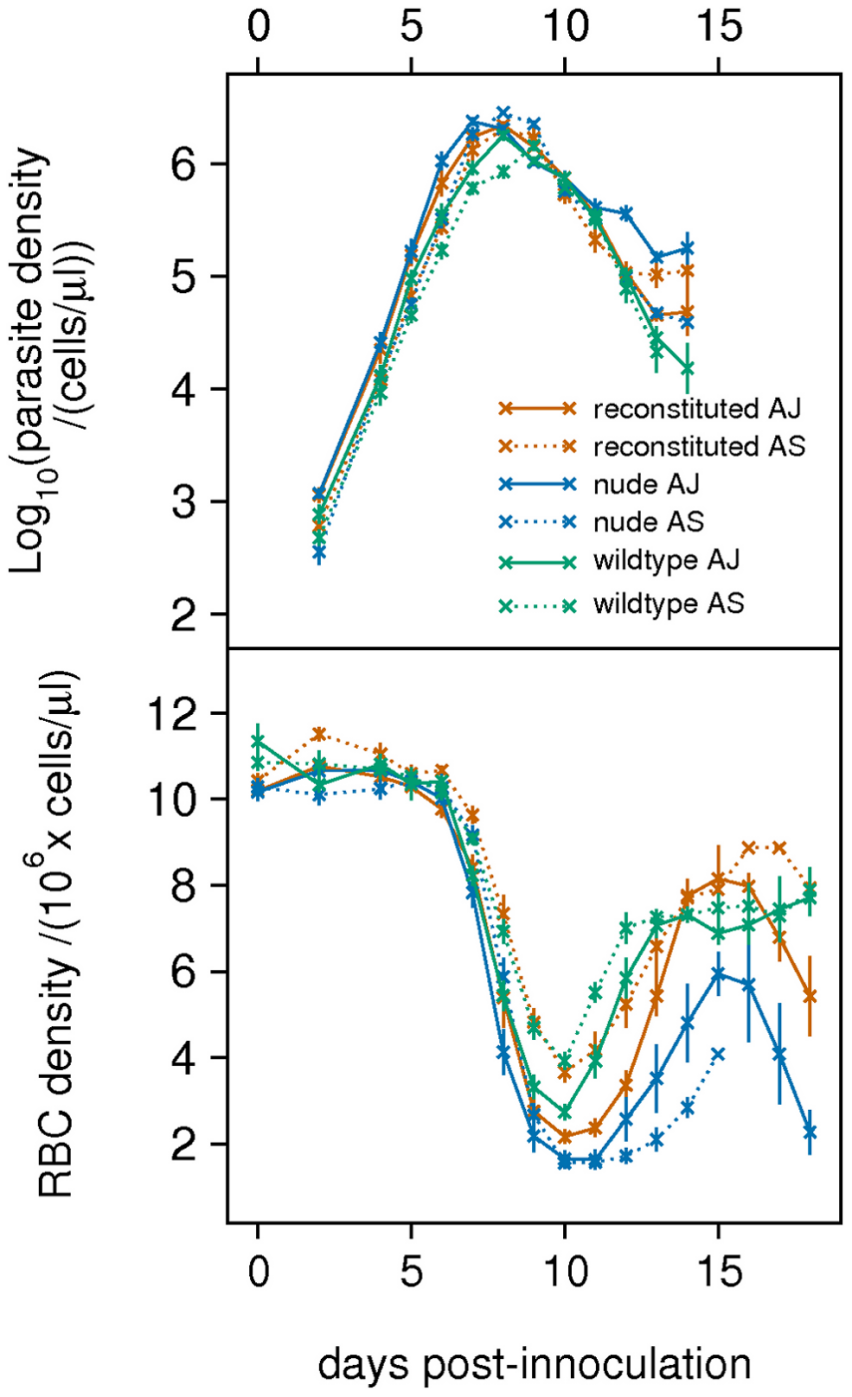
Parasite and RBC dynamics. Data on parasite density and RBC density, averaged across mice from individual treatments (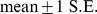).

The averaged dynamics of each treatment are shown in Fig. 1. The AJ clone (solid line) does not show the normal higher peak density compared to the AS clone (dotted line); however, the AJ clone exhibits a higher density during the exponential growth phase compared to AS. Parasite density tends to level off in reconstituted and nude mice from day 12, but continues declining in the wildtype, presumably because of a stronger immune response in these mice.

In reconstituted and wildtype mice, the AJ clone causes greater anaemia than the AS clone. All three mouse phenotypes show an earlier drop in RBC density from days 6–8 when infected with AJ compared to AS. The recovery of RBC density in nude mice is weaker than in reconstituted and wildtype mice. We discuss these observations below in relation to inferences from the model fitting.

### The mathematical model

In *Plasmodium chabaudi*, pRBCs rupture synchronously every 24 hours, releasing on average 6–8 parasites (merozoites) into the bloodstream [40]. These newly released merozoites infect further RBCs and the cycle repeats. The rupture of pRBCS (schizogony) occurs at approximately midnight under normal lighting conditions [41].

We use a discrete-time formulation to model the dynamics, where each time step corresponds to a single day. The start of day 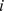 is defined as the point immediately following rupture of pRBCs, before any infection has occurred (i.e., the point at which merozoites are released into the bloodstream). The densities of merozoites and uRBCs at the start of day 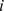 are denoted 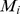 and 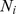, respectively. We assume that the processes determining RBC density occur on two non-overlapping timescales. The first corresponds to the short infection phase during which merozoites infect RBCs, which occurs within a few minutes following schizogony. The second and subsequent timescale (the remainder of the day) corresponds to the RBC turnover phase: here the parasites replicate within pRBCs, new uRBCs are released into the bloodstream and, if active, the (non-merozoite) immune responses clear pRBCs and uRBCs. At the end of the RBC turnover phase, surviving pRBCs rupture and release new merozoites.

#### The infection phase

The infection dynamics at the start of day 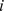 are modelled in continuous time 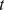, using a formulation based on [14]. Let 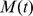, 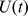, 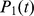 and 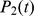 denote, respectively, the densities of merozoites, uRBCs, pRBCs containing a single parasite, and pRBCs containing multiple parasites. The variables' dependence on 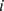 is dropped for clarity. The dynamics are then described by the following system of differential equations:
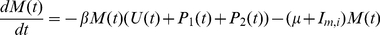
(1)

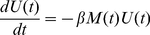
(2)

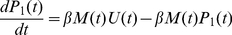
(3)

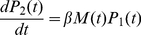
(4)Here 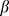 is the rate at which merozoites infect RBCs. The constant 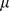 gives the decay rate of merozoites due to natural mortality and loss from the circulation. The function 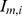 is the immune-mediated clearance rate of merozoites on day 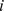, which is assumed constant over the short infection phase. Time 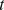 has units of seconds, reflecting the rapid infection of RBCs.

The initial densities are defined as: 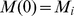, 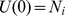 and 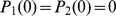. The total number of RBCs, 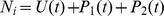, remains constant over this timespan. An explicit solution to equation (1) is obtained by integrating with respect to 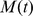, i.e.,
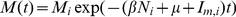
(5)


As noted above, infection occurs over a relatively short timescale compared to the other processes determining the RBC densities. We therefore assume that the densities at the end of the infection phase are given in the limit as 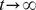. In this limit, the density of merozoites tends to zero (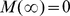).

In allowing for the possibility of multiple infections, we introduce two further variables. Let 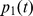 and 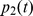 denote the total densities of parasites (merozoites) within singly-parasitised and multiply-parasitised RBCs, respectively. Note that, by definition, 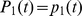 and 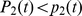.

The RBC and parasite densities following infection can now be derived. Once released into the bloodstream, a given merozoite will either infect a RBC or be removed from the circulation (due to natural decay or the activity of the merozoite-targeting immune response). These processes occur at rates 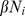, 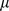, and 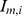, respectively. A given merozoite therefore has probability 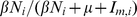 of infecting a RBC. Multiplying this probability by the initial density of merozoites, and dividing by the total RBC density, gives the average number of parasites per RBC (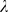):
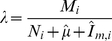
(6)


The substitutions 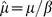 and 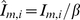 are made because 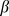 and 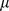 are non-identifiable (i.e., we cannot estimate their values separately).

Since infections occur independently, the probability of a given RBC being infected by 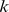 merozoites is Poisson-distributed with parameter 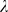, i.e., 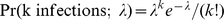. The uRBC, pRBC and parasite densities at the end of the infection phase are therefore:
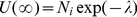
(7)

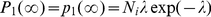
(8)

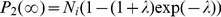
(9)

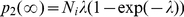
(10)


The density of parasites within multiply-parasitised RBCs is obtained by noting that the total parasite density equals 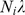.

#### The RBC turnover phase

The RBC turnover dynamics occur after the infection dynamics on each day 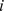. They are modelled in continuous time 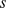, which has units of days. Let 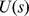, 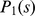 and 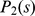 denote, respectively, the densities of uRBCs, singly-parasitised RBCs and multiply-parasitised RBCs on this timescale. Similarly, let 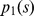 and 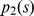 denote, respectively, the densities of parasites within singly-parasitised and multiply-parasitised RBCs. Again, we drop the dependence on 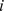 for clarity.

In the absence of infection, RBCs are lost through natural mortality and gained through the production of new cells (erythropoiesis). We take RBC decay as 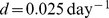 [42], [43]. This implies that the baseline erythropoiesis rate, needed to replenish the lost RBCs, is 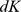, where 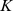 is the normal RBC density.

Erythropoiesis may be upregulated in response to the anaemia caused by the infection. We assume that erythropoiesis upregulates 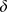 days after initial innoculation. The level of upregulation on day 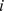 is linearly proportional to the difference between the normal RBC density, 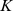, and the RBC density 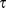 days earlier, 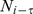. The parameter 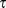 measures the lag in feedback between RBC density and the level of erythropoiesis. The upregulation of erythropoiesis is then given by 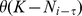, where 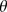 gives the proportion of the RBC deficit that is recovered.

We assume that multiply-parasitised RBCs have an additional mortality rate, 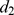. This allows for the possibility that individual RBCs infected with more than one parasite may be exploited to such an extent that the cell dies before the parasite is able to reproduce (i.e., prior to schizogony). The increased mortality rate could conceivably vary between 0 and 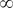 as we have no prior evidence to constrain it. We therefore re-parameterise using 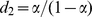, with a prior on 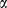 of Uniform(0, 1).

The immune-mediated clearance rates of pRBCs and uRBCs on day 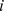 are given by the functions 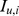 and 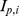 respectively. As such, we assume that they change from one day to the next, but remain constant within a single day. This, and the relationship between erythropoiesis and 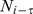, are simplifying assumptions that allow us to derive analytical solutions to our equations. Using continuous-time functions would require numerical solutions of delay differential equations which would be computationally prohibitive. A consequence of these assumptions is that immune and erythropoietic rate parameter estimates may be biased. This is of no practical significance in our analysis because we are interested in relative differences in immune and erythropoietic responses across experimental treatments, rather than absolute values.

Given the above assumptions, the dynamics of the RBC turnover phase are described by the following system of differential equations:
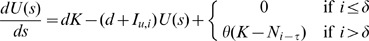
(11)

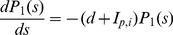
(12)

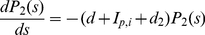
(13)

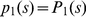
(14)

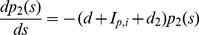
(15)The solution of this system of equations is derived in the Supporting Information (Text S1).

#### Rupture of pRBCs

The final event on day 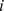 is the rupture of pRBCs and release of merozoites. At this point each surviving parasite produces 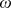 merozoites and the pRBC density returns to zero. The uRBC density on the 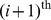 day is equal to 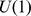, given as:
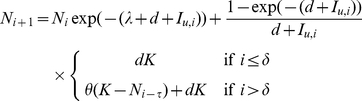
(16)The merozoite density on the 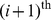 day is equal to 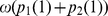:
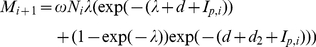
(17)


Equations (16)–(17) define the model for updating the uRBC and merozoite densities immediately following rupture. At this time (about 00:00 hrs) the parasite density is equal to the merozoite density. In the experiment, however, RBC and parasite densities were measured at approximately 08:00 hrs, roughly 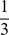 of the time between successive rupture events. We therefore fit the model predictions for RBC and parasite densities during the RBC turnover phase at the point when 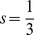.

The mice were innoculated intraperitoneally with 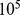 singly-parasitised RBCs at 08:00 hrs on day 0. Although all mice received this same initial dose, we considered that not all of the pRBCs might enter the bloodstream; we therefore fit the initial density of pRBCs, 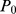, to the data. The initial RBC density was assumed to be the normal RBC concentration, 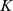. The full list of variables, functions and parameters is given in Table 1.

**Table 1:** Variables, functions and parameters used in the models.

**Independent variables**		
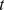	time	second
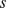	time	day
**Dependent variables**		
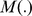	density of merozoites	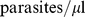
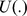	density of uRBCs	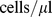
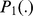	density of singly-parasitised RBCs	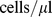
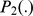	density of multiply-parasitised RBCs	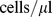
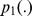	parasite density (singly-parasitised RBCs)	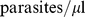
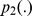	parasite density (multiply-parasitised RBCs)	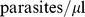
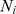	total RBC density on day 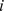	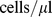
**Functions**		
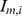	immune-mediated clearance rate of merozoites on day 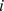	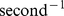
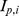	immune-mediated clearance rate of pRBCs on day 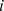	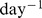
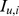	immune-mediated clearance rate of uRBCs on day 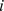	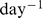
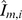	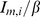	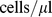
**Parameters**		
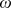	burst size	parasites
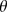	rate of upregulation of erythropoiesis	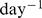
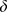	time delay before upregulation	day
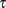	time lag in erythropoiesis	day
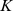	normal RBC density	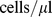
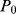	initial parasite density	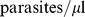
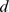	natural death rate of RBCs	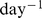
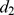	increased death rate of multiply-parasitised RBCs	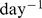
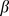	infection rate	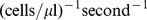
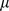	natural loss rate of merozoites	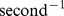
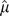	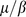	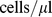
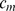	maximum level of merozoite-targeting immunity	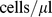
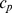	maximum clearance rate of pRBC-targeting immunity	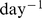
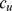	maximum clearance rate of uRBC-targeting immunity	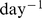
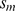	start day of merozoite-targeting immunity	day
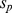	start day of pRBC-targeting immunity	day
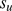	start day of uRBC-targeting immunity	day
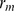	rise time of merozoite-targeting immunity	day
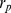	rise time of pRBC-targeting immunity	day
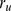	rise time of uRBC-targeting immunity	day
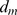	duration of merozoite-targeting immunity	day
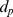	duration of pRBC-targeting immunity	day
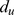	duration of uRBC-targeting immunity	day

#### Immune-mediated clearance rates

We model the immune response as three distinct components that clear merozoites, pRBCs and uRBCs, respectively. On a given day 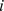, the functions 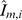, 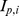 and 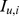 give the immune-mediated clearance rates of merozoites, pRBCs and uRBCs, respectively. The merozoite targeting response operates during the infection phase, while the pRBC and uRBC targeting responses operate during the RBC turnover phase.

An immune component is defined in terms of four parameters: the day of initial activation (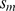, 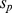, 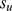), the maximum clearance rate (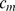, 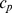, 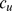), the time (measured in days) taken to reach maximum clearance rate (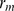, 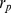, 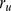), and the duration in days of the immune response (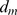, 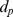, 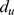). Initially, the clearance rates of all three immune responses are set to zero. When an immune response is activated, the clearance rate increases to its maximum level over a number of days. Once the given duration has elapsed, as measured from the start day, the clearance rate returns to zero. The expressions for the three immune responses are
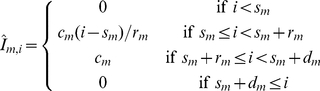
(18)

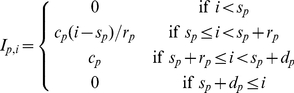
(19)

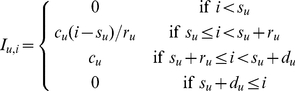
(20)


### Tested hypotheses

Using the statistical algorithm described in the Supporting Information (Text S1, Figure S1 and Figure S2), we fit to the observed data on RBC and parasite densities. The fitted parameters are given in Table 2, along with their prior distributions. The prior distributions were based either on values taken from the literature, or approximate estimates obtained before the main model-fitting (see Text S1 for details).

**Table 2:** Parameters and their prior distributions.

Parameter	Prior distribution	Source
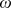	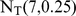	[40]
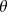	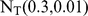	[8], [14]
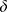	U(0, 12)	[30]
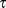	U(0, 6)	[14], [58]
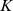	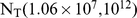	
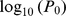	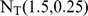	
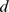	0.025	[42], [43]
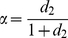	U(0, 1)	
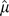	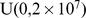	
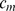	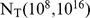	
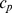 , 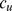	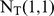	
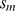 , 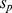 , 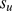	U(0, 19)	
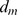 , 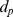 , 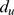	U(0, 19)	
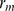 , 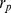 , 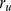	U(0, 19)	

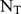
 is a normal distribution truncated at 0.

Our aim is to find a set of minimal adequate models which explain the data well and contain as few parameters as possible. We take as our baseline the model described above. This is denoted 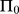 and contains 20 fitted parameters. We developed a set of nested and non-nested models in which specific assumptions are made about the immune and erythropoietic responses (outlined in Table 3). All models were fitted to the data. Each model fit was then evaluated relative to that of the baseline. The model fits were compared using Bayes' factors, which naturally penalise overfitting [44] (refer to Text S1 for further details on measurement error, the likelihood function, model fitting, assessment and comparison).

**Table 3:** Description of tested models.

Model	Description	parameters affected
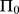	baseline model	
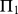	no merozoite clearance	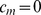
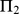	no pRBC clearance	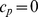
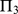	no uRBC clearance	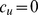
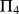	synchronised pRBC and uRBC clearance	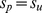 , 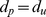 , 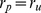
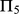	synchronised merozoite and uRBC clearance	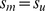 , 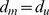 , 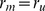
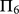	synchronised merozoite and pRBC clearance	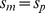 , 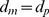 , 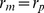
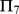	pRBC clearance after peak parasite density	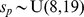
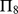	uRBC clearance after peak parasite density	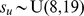
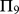	merozoite clearance after peak parasite density	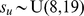
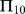	no RBC upregulation	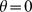
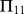	no delay in upregulation of erythropoiesis	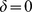
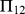	no time lag in erythropoieses	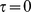
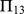	no delay or time lag in erythropoiesis	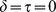
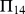	no loss of multiply-parasitised RBCs	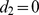
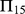	multiply-parasitised RBCs produce only 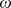 merozoites	

We estimate parameters separately for each mouse, rather than across treatments. Even inbred mice are phenotypically different, and these differences result in variability in parasite and RBC dynamics. Immune responses are significant sources of variability *in vivo*; but we might also expect variation between mice in parameters such as infection rate, because of the multifactorial nature of such processes which involve the interaction of many host and parasite proteins. We therefore make no assumption about which parameters are invariant across mice and instead estimate parameters separately for each mouse. This method allows us to infer parameter (and hence process) variability within and between treatments from the posterior estimates.

#### Removal of immune components

We investigated removing individual components of the immune response (i.e., the merozoite, pRBC or uRBC targeting components). The remaining two components were assumed to function as normal.

#### Synchronisation of immune components

This involved setting the same start, rise and duration times for different immune components. Thus, synchronisation of the pRBC and uRBC targeting arms corresponded to 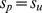, 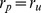 and 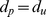. Note that the synchronised components generally have different maximum levels (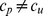).

#### Control of peak parasite density

All three immune components are included in the model and function independently. However, the start time of a given component is restricted to occur after the initial peak of parasite density: the earliest this immune component can activate is day 8 post-innoculation. The number of parameters is the same as in the baseline model, but a restriction is placed on the prior for immune start time.

#### Upregulation of erythropoiesis

We considered omitting the upregulated erythropoietic response. In this case the parameter 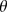 is set to zero, which implies 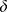 and 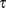 are unnecessary (see equation (11)). We also separately investigated the effect of omitting the delay, or the lag in feedback time, or both.

#### Mortality of multiply-parasitised RBCs

In the baseline model, multiply-parasitised RBCs have a higher mortality rate than singly-parasitised RBCs. We examined a model where multiply-parasitised and singly-parasitised RBCs have the same mortality rate (i.e., setting the increased mortality rate, 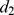, to zero).

#### Number of merozoites produced per multiply-parasitised RBC

In the baseline model, multiply-parasitised RBCs produce merozoites relative to the number of parasites that infect them (each parasite produces 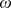 merozoites upon schizogony). However, it is possible that there is some constraint on the number of merozoites due to host resources. We therefore examined a model where each multiply-parasitised RBC produces only 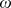 merozoites, i.e., 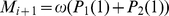.

## Results

### Assessment of the baseline model

**Figure 2:**
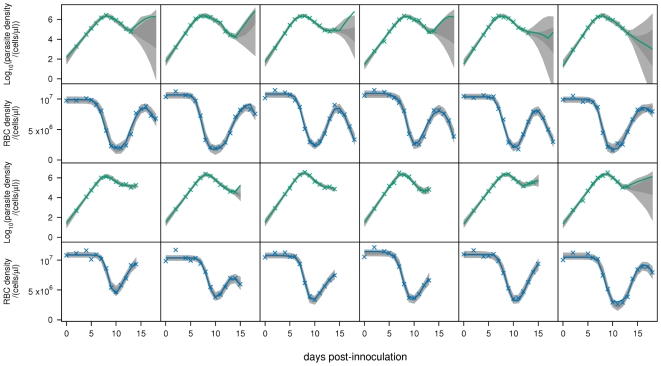
Model fits. Fits of baseline model 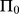 to parasite density and RBC density for reconstituted mice infected with the AJ strain (top panels), or the AS strain (bottom panels). Crosses are data. Light-grey regions correspond to 95% posterior predictive intervals (PPI); dark-grey regions correspond to 50% PPIs. The solid lines give the best-fit (posterior mode) solutions.

**Figure 3 pcbi-1000946-g003:**
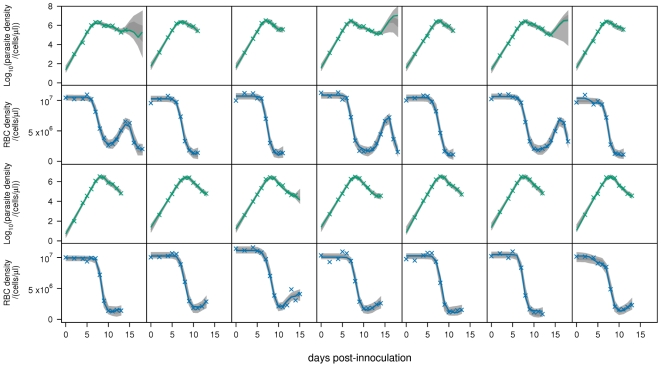
Model fits. Fits of baseline model 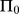 to parasite density and RBC density for nude mice infected with the AJ strain (top panels), or the AS strain (bottom panels). Crosses are data. Light-grey regions correspond to 95% posterior predictive intervals (PPI); dark-grey regions correspond to 50% PPIs. The solid lines give the best-fit (posterior mode) solutions.

**Figure 4:**
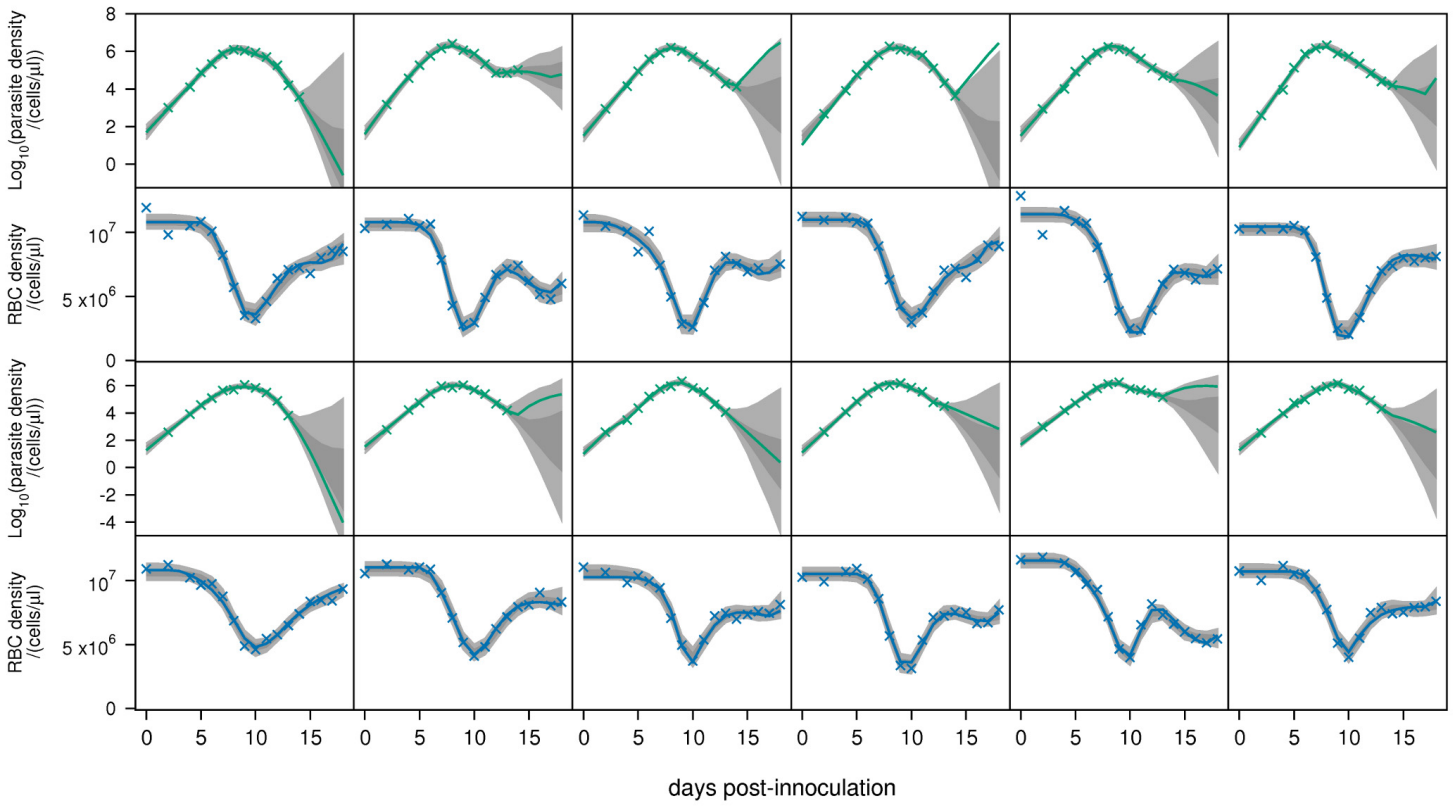
Model fits. Fits of baseline model 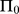 to parasite density and RBC density for wildtype mice infected with the AJ strain (top panels), or the AS strain (bottom panels). Crosses are data. Light-grey regions correspond to 95% posterior predictive intervals (PPI); dark-grey regions correspond to 50% PPIs. The solid lines give the best-fit (posterior mode) solutions.

We begin by analysing the baseline model, 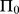. The fits to all mice are shown in Figs. 2–4. Note that the posterior predictive interval (PPI) of the dynamics widens from around day 15 for some mice because of the lack of data.

**Figure 5:**
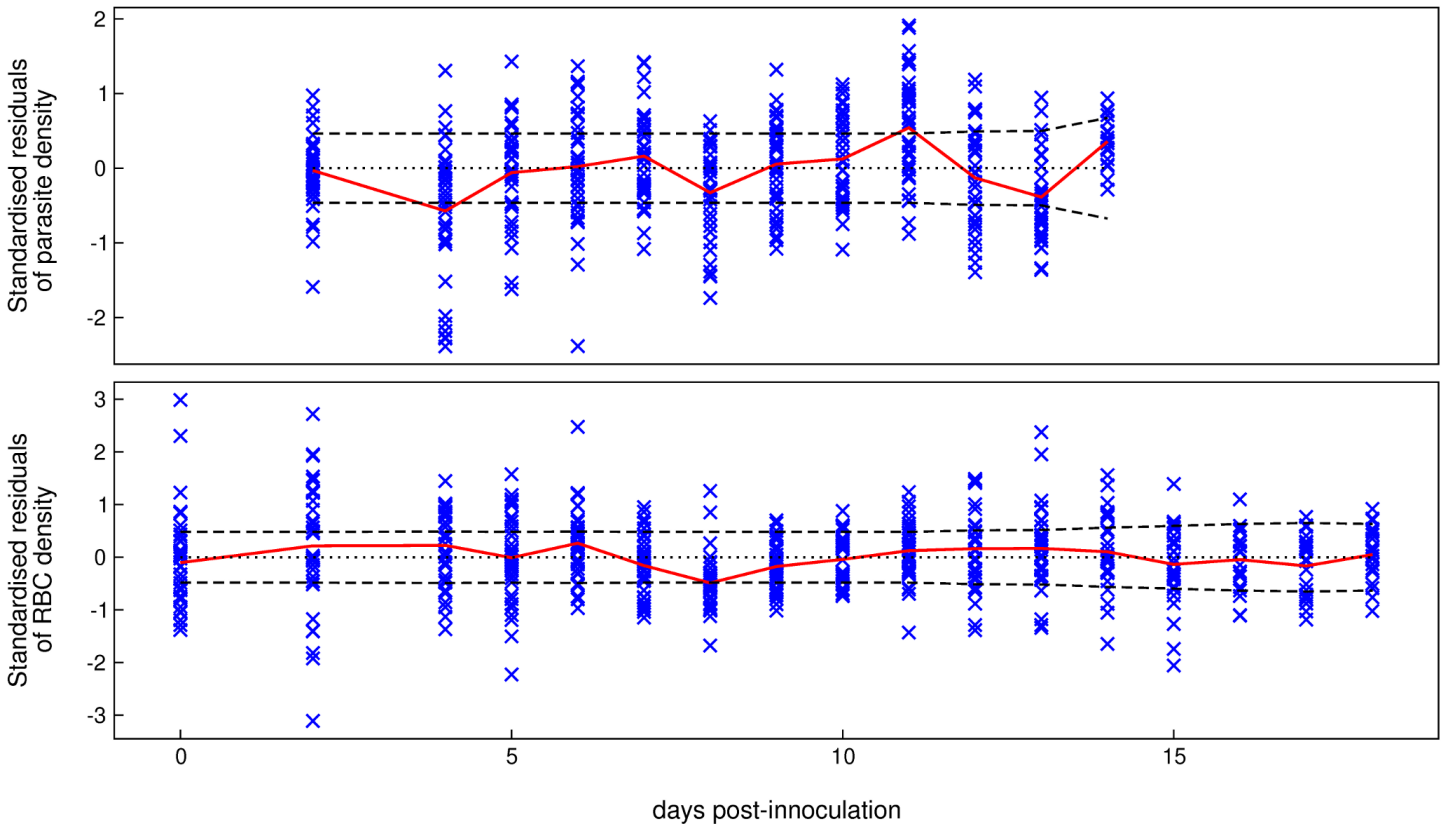
Standardised residuals. Assessment of baseline model fits to all mouse data by standardised residuals. Top panel: parasite density, bottom panel: RBC density. Each cross represents the standardised residual of a time point for an individual mouse. These have an approximately normal distribution with mean 0 and variance 1. The solid red line joins the means of the standardised residuals at each time point. The dashed lines delimit the 95% predictive interval for the expected mean standardised residual for the same number of residuals as the data, Bonferroni corrected for the number of time points (see Text S1 for details). The model systematically overestimates the data when the red line lies below the 95% predictive interval, and underestimates the data when it lies above this interval. The y-axis is scaled in units of standard deviations.

The model fits appear to adequately explain the data. A more rigorous assessment of the model fits is attained by plotting the overlaid standardised residuals for parasite and RBC densities. Fig. 5 shows the standardised residuals (the blue crosses) for all mice across the six treatments. Poor fits are suggested by outlying and serially correlated residuals.

The fits to parasite density are accurate but not perfect. There are no outliers, but the model tends to overestimate parasite density on day 4. This is unexpected because we expect parasite density to be growing exponentially during this time, and indeed this is how the model behaves. This discrepancy suggests that parasite density may initially grow at a slower than exponential rate. Also, the model tends to underestimate parasite density on day 11. Currently, we have no explanation for this. The fits to RBC density are accurate, except on day 8 where RBC density is marginally overestimated. Therefore, the model may not be correctly capturing the trough in RBC density.

### Analysis of hypotheses

We calculated the Bayes' factors of models 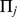, for 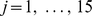, relative to the baseline model 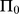. We adopt the scale of interpretation for Bayes' factors proposed by Jeffreys [45] and reproduced in Table 4. For ease of interpretation, the Bayes' factors are converted to deciBans; i.e., 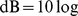
(Bayes' factor). Bayes' factors were calculated for each mouse, giving a total of 38 values for each model comparison. The full list of Bayes' factors is given in Table S1 and Table S2. For conciseness and clarity, we report for each model: (i) the sum of the deciBans for all mice within each treatment, and (ii) the sum of the deciBans for all mice across treatments (Table 5). We also report the standard error of the deciBans. Errors occur because deciBans are estimated from a finite sample of the posterior distribution. Our inferences are conservative; thus, we interpret a deciBan of 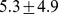 as “barely worth mentioning” (see Table 4).

**Table 4:** Scale of interpretation of Bayes' factors.

dB	Evidence against model 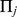
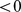	Negative (supports 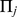 )
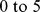	Barely worth mentioning
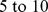	Substantial
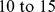	Strong
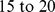	Very strong
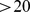	Decisive

Values are in deciBans (dB).

**Table 5:** Comparison of each model to the baseline model 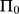 using Bayes’ factors.

	Model				
Treatment	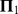	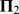	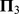	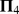	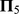
Reconstituted AJ	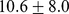	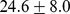	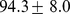	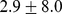	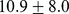
Reconstituted AS	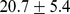	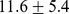	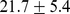	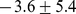	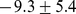
Nude AJ	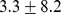	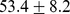	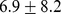	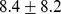	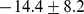
Nude AS	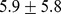	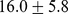	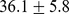	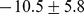	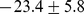
Wildtype AJ	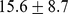	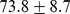	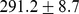	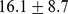	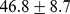
Wildtype AS	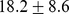	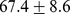	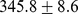	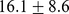	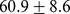
All	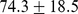	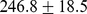	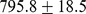	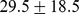	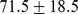

Values are 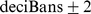 standard errors of the deciBans for each treatment and all treatments.

Statistical comparison of parameter values between treatments was performed using Analysis of Variance (ANOVA) in the R statistical package [46]. The method was as follows. For a given parameter, we first took the mean of the posterior distribution, 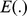, for each individual mouse. The mean for a given treatment, 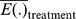, was then calculated as the average of the posterior mean values taken across all mice in the treatment.

#### Removal of immune components

We tested which of the immune components are necessary to explain our data. There is substantial to decisive evidence for immune-mediated clearance of pRBCs (Table 5, 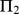). The evidence is weakest in AS-infected reconstituted mice (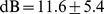) and AS-infected nude mice (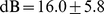), and strongest in wildtype mice (AJ: 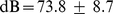, AS: 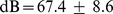). This may simply reflect the shorter time series in the reconstituted and nude mice, or alternatively, may indicate a stronger immune response in the wildtype. Overall, the evidence for pRBC clearance is decisive (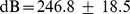).

We then considered the evidence for immune-mediated clearance of uRBCs (Table 5, 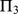). In all treatments except the AJ-infected nude mice, there is very strong to decisive evidence for this mechanism. The most evidence is in wildtype mice (AJ: 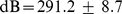, AS: 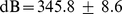). Overall, the evidence for uRBC clearance is decisive (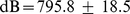).

Next, we considered the evidence for immune-mediated clearance of merozoites (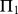, Table 5). There is no evidence in nude mice (AJ: 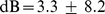, AS: 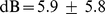), or AJ-infected reconstituted mice (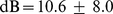). However, there is substantial evidence in wildtype mice (AJ: 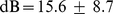, AS: 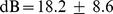), and very strong evidence in AS-infected reconstituted mice (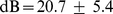). Overall, the evidence for this immune mechanism is decisive (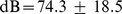). It is notable that the Bayes' factors for immune-mediated merozoite clearance are, on the whole, much lower than those for pRBC clearance and uRBC clearance. This suggests that, although immune-mediated merozoite clearance definitely occurs, its effect on the RBC and parasite dynamics is relatively weak compared to that of the other immune components.

**Figure 6:**
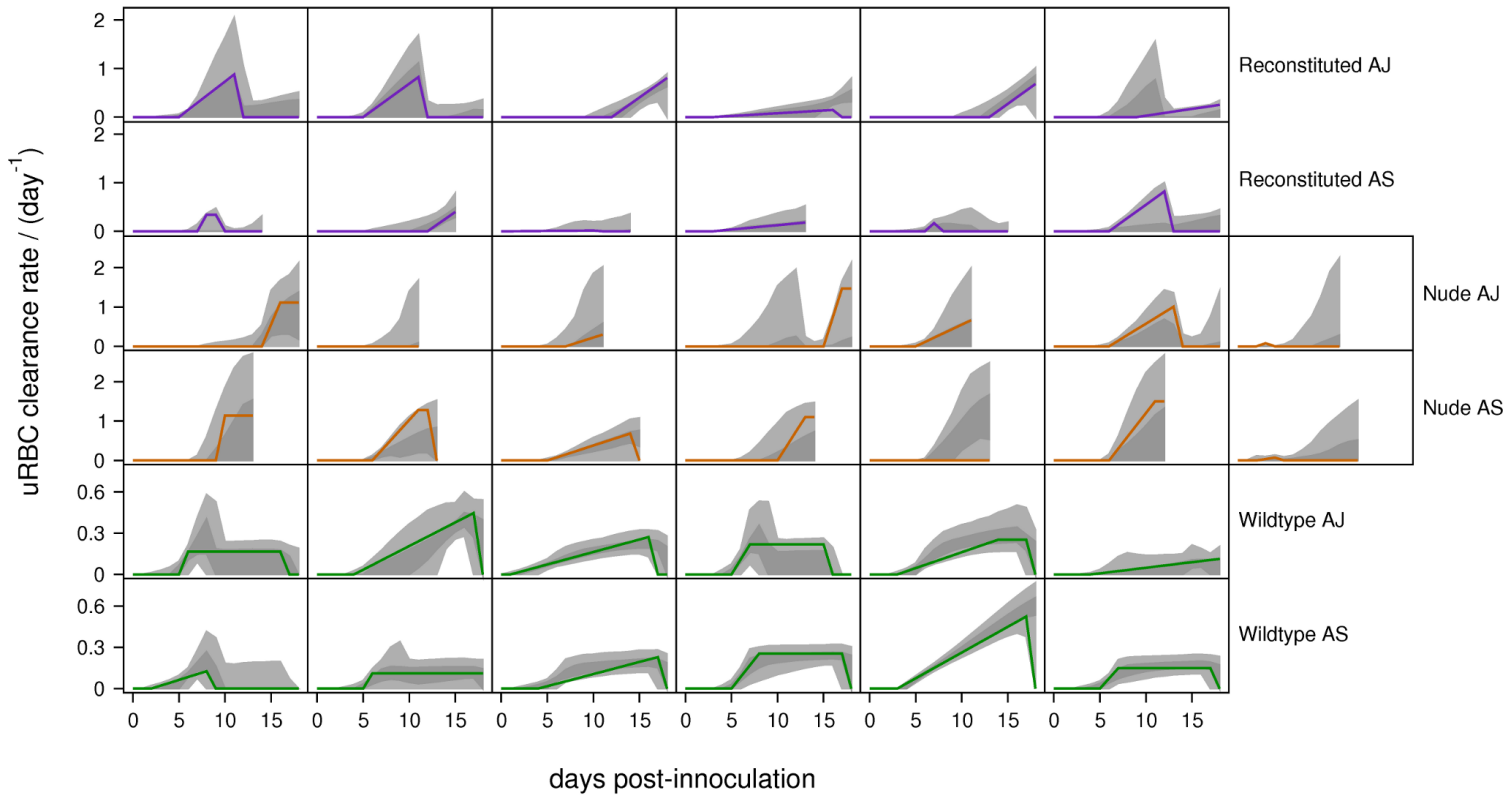
Predicted uRBC clearance rates. Posterior predictive intervals of immune-mediated uRBC clearance rates for the baseline model. Light-grey regions correspond to 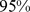 PPIs; dark grey regions correspond to 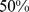 PPIs. The solid lines give the best-fit (posterior mode) solutions. Note the different scales for reconstituted, nude and wildtype mice.

**Figure 7:**
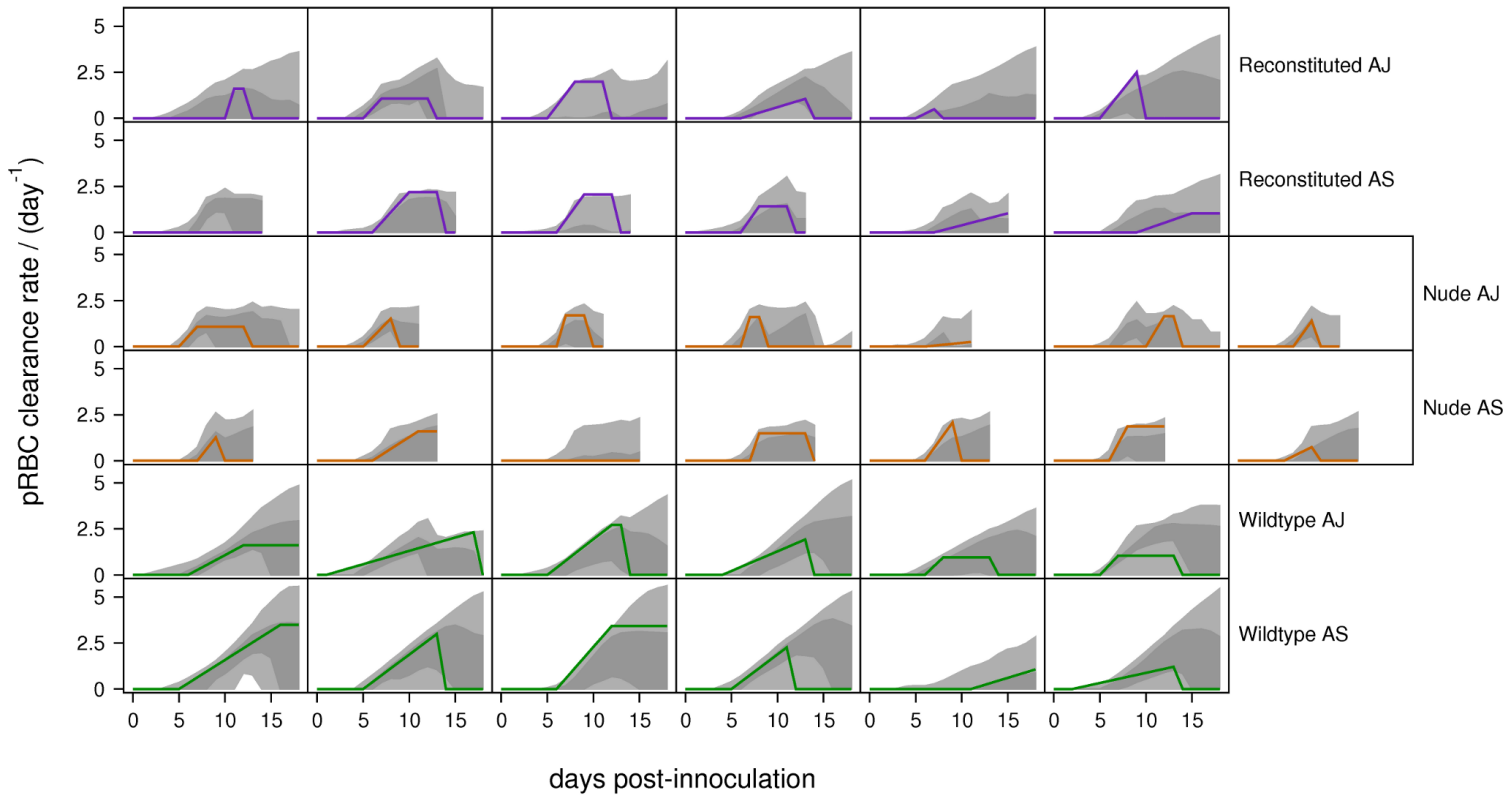
Predicted pRBC clearance rates. Posterior predictive intervals of immune-mediated pRBC clearance rates for the baseline model. Light-grey regions correspond to 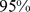 PPIs; dark grey regions correspond to 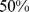 PPIs. The solid lines give the best-fit (posterior mode) solutions.

The 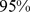 PPIs of the immune-mediated uRBC and pRBC clearance rates are shown in Figs. 6 and 7. For the wildtype mice (for which we have the most data), the PPIs of the uRBC clearance rates do not contain zero (Fig. 6). By contrast, in all of the nude and most of the reconstituted mice, the 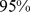 PPIs of the uRBC clearance rates always contain zero. This shows the crucial importance of uRBC clearance in the wildtype mice, as reflected in the very high Bayes' factors (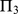, Table 5). The 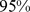 PPIs of the pRBC clearance rates contain zero for all except one of the mice (Fig. 7). We believe this is due to the fact that merozoite clearance, by directly targeting the parasite, is able to compensate for the absence of this mechanism.

Wildtype mice were found to have lower maximum uRBC clearance rates than reconstituted mice (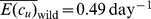, 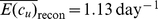, 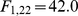, 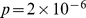). In turn, the reconstituted mice have lower maximum uRBC clearance rates than nude mice (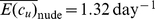, 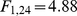, 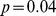). These results suggest that the higher immunocompetence of wildtype mice (and to a lesser degree, reconstituted mice) is associated with weaker clearance of uRBCs.

#### Synchronisation of immune components

We considered whether the pRBC and uRBC immune components could be synchronised, thus reflecting a common effector mechanism (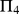, Table 5). There is substantial evidence against synchronisation in wildtype mice (AJ: 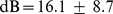, AS: 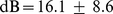). The other treatment groups provide no significant evidence either for or against synchronisation. Overall, there is strong evidence that the uRBC and pRBC targeting components are not synchronous (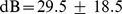). However, the evidence against synchronisation of merozoite and uRBC clearance, or synchronisation of merozoite and pRBC clearance is decisive (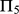 and 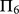, Table 5). This suggests that, although they are not synchronous, the temporal patterns of pRBC and uRBC clearance in *P. chabaudi* are closer to each other than to the temporal pattern of merozoite clearance.

#### Control of peak parasite density

We investigated if the immune-mediated responses targeting merozoites, pRBCs and uRBCs are associated with control of the initial peak of parasite density. We restricted the immune responses to occur only after the initial peak. Since the peak occurs on days 7–9 post-innoculation, the priors on 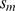, 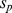 and 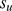 were therefore restricted to Uniform(8, 19). Thus, the initial clearance could not begin until day 9 at the earliest.

Across the treatments, there is substantial to decisive evidence that pRBC clearance is associated with control of the peak of parasite density (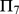, Table 5). This hypothesis is supported in all three phenotypes (wildtype, reconstituted, nude). The evidence that uRBC clearance is associated with control of the initial peak differs greatly across the treatments (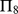, Table 5): there is decisive evidence in wildtype mice, and strong evidence in AS-infected nude mice, but no evidence in reconstituted mice or AJ-infected nude mice. There is very strong evidence that merozoite clearance is associated with control of the peak in AS-infected reconstituted mice, but no evidence in any of the other treatments (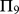, Table 5).

#### Upregulation of erythropoiesis

**Figure 8:**
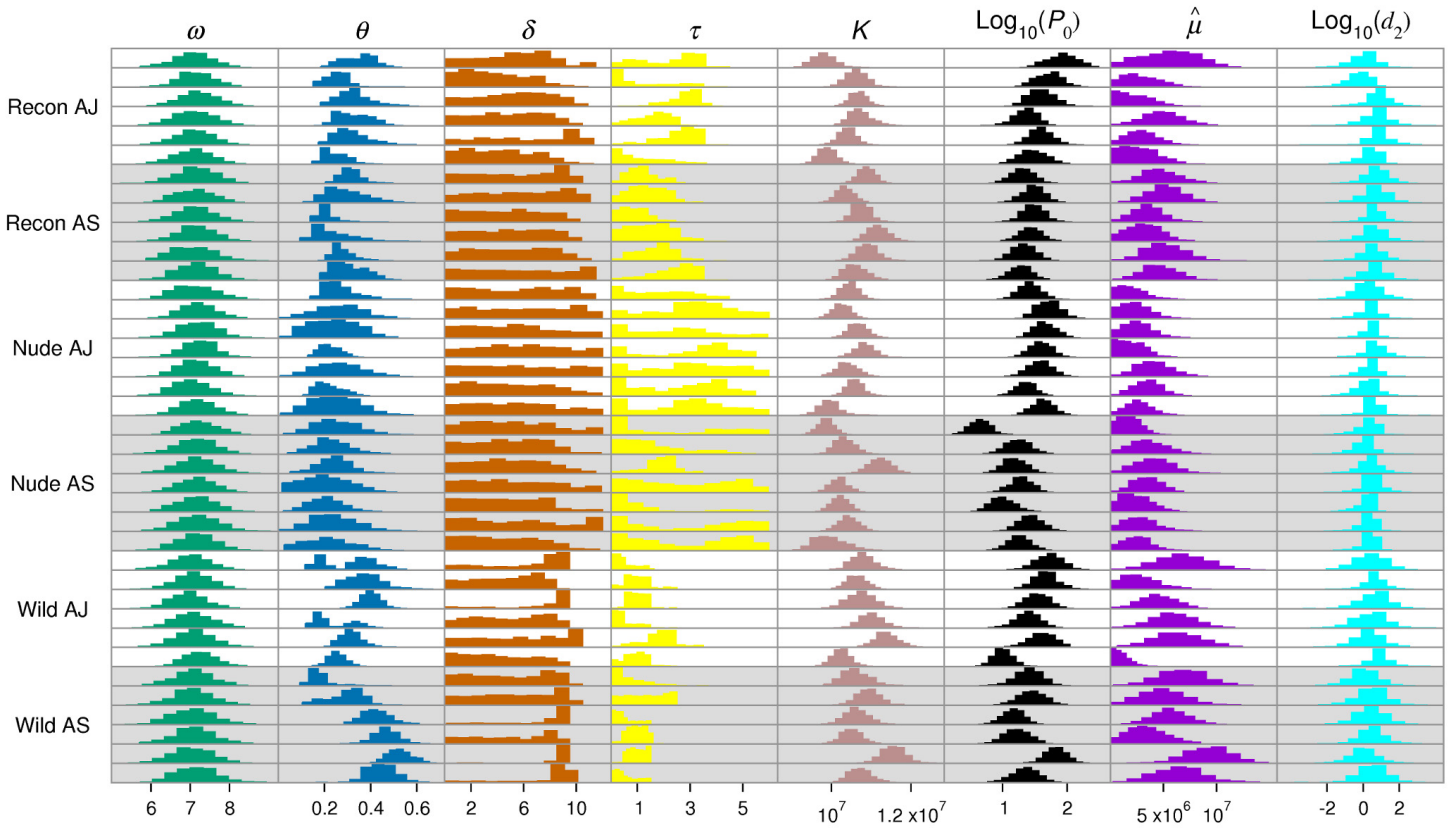
Parameter estimates. Marginal distributions of the fitted parameters for the baseline model. Histograms are for individual mice. Panels with white backgrounds correspond to AJ-infected mice; panels with grey backgrounds correspond to AS-infected mice.

We investigated if erythropoiesis is upregulated above its baseline level in response to anaemia, and if so, the form of this upregulation. There is decisive evidence for upregulation of erythropoiesis across all treatments (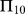, Table 5). The marginal posterior distributions for the level of upregulation, 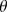, are shown in Fig. 8. The level of upregulation is significantly different in nude mice compared to wildtype and reconstituted mice (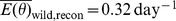, 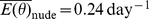, 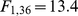, 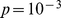).

In five of the six treatments there is no evidence for a delay prior to upregulation (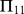, Table 5). The exception is in AS-infected wildtype mice, which give strong evidence for a delay (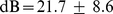). The marginal posterior distributions of 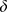 indicate a delay of around 9 days in these mice (Fig. 8).

The AJ-infected reconstituted mice provide strong evidence for a time lag in the feedback between RBC density and the erythropoietic response (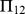, 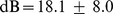, Table 5). There is no evidence for a time lag in any of the other treatments; in fact, including a time lag causes overfitting of the model in the nude and AS-infected wildtype treatments (indicated by the negative deciBan values).

We also investigated a model which omitted both the time delay before upregulation, and the lag in feedback in the erythropoietic response (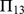, Table 5). The strongly negative deciBans for the nude treatments suggest overfitting in these mice. The evidence in wildtype and AJ-infected reconstituted mice is strong to decisive for at least one process (i.e., it suggests either a delay before upregulation, or a lag in feedback time).

#### Mortality of multiply-parasitised RBCs

We investigated whether multiply-parasitised RBCs are killed before schizogony occurs (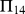, Table 5). Overall, there is decisive evidence that multiply-parasitised cells experience a higher mortality rate (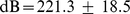). The fitted value of 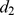, averaged across all mice (Fig. 8), is 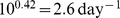, which translates into a half-life of about 6.4 hrs. However, the marginal posteriors vary from 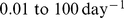, so the data do not allow us to estimate a precise value.

#### Number of merozoites produced per multiply-parasitised RBC

We investigated whether, in multiply-parasitised RBCs that survive to schizogony, each parasite produces 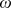 merozoites, or whether only a single parasite survives to produce 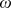 merozoites (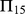, Table 5). In terms of the Bayes' factors, there is no evidence to distinguish between these two hypotheses (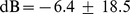). This may be due to the higher mortality rate of multiply-parasitised RBCs. According to this explanation, because only a few of the multiply-parasitised cells survive to reproduce, the overall effect of these cells producing a higher number of parasites is negligible. In this case, we cannot draw a conclusion either way.

### Other inferences

#### Difference between the AS and AJ clones

The AJ strain is known to be a more virulent clone than AS. We explored the potential reasons for this. There was no difference between the clones in terms of the maximum immune-mediated pRBC clearance rate (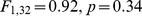), or the maximum immune-mediated uRBC clearance rate (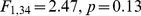). Neither was there a difference in the additional mortality rate of multiply-parasitised cells (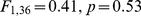). The posterior means of the transmission parameter, 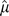, also did not differ between the clones (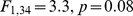).

Interestingly, the initial parasite density was found to be higher in the AJ clone compared to AS (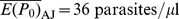, 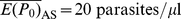, 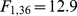, 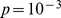; Fig. 8). Although the infective dose was the same for all mice, a higher initial density for the AJ clone could have arisen through experimental error; alternatively, it could be due to the AJ clone being better at entering the bloodstream from the peritoneum. Either hypothesis could explain the higher AJ densities during the exponential growth phase, and why the AJ clone causes an earlier drop in RBC density (Fig. 1).

#### Difference between the phenotypes

If we consider the marginal posterior distributions for burst size, 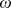, it is clear that these do not differ from the prior distribution of Normal(7, 1) across any of the mice (Fig. 8). This implies that the data contain no more information about burst size than our prior knowledge.

We observed a difference between the phenotypes in the transmission parameter, 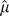. The mean value of this parameter was significantly higher in wildtype and reconstituted mice compared to nude mice (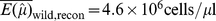, 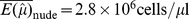, 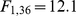, 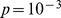). As 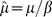, this suggests that either the background mortality rate of merozoites is higher, or that the RBC infection rate is lower, in wildtype and reconstituted mice than in nude mice.

#### RBC dynamics after the peak of parasite density

After day 12, the 95% PPIs of the dynamics show that parasite densities can increase or decrease (Figs. 2–4). This is due to a lack of data describing parasite density, and implies that infection has a marginal or negligible effect on the RBC dynamics at this stage.

We predict that immune-mediated uRBC clearance is the major determinant of RBC dynamics over days 12–18. This can be seen by comparing the RBC dynamics (Figs. 2–4) and the predicted uRBC clearance rates (Fig. 7). The correlations are particularly clear in wildtype (AS and AJ) and reconstituted (AJ) mice. Whether RBC density rises, plateaus or declines after day 12, depends on whether the uRBC clearance rate drops to zero, plateaus or rises, respectively.

## Discussion

The aim of this paper was to provide a quantitative assessment of the immune and erythropoietic responses in *Plasmodium chabaudi* infections. Hypotheses were drawn from experimental data and the mathematical modelling literature. These were fit to data on malaria infected mice using a Bayesian statistical framework. Crucially, by quantifying the experimental error, we were able to provide a rigorous assessment of the model fit. In particular, we were able to evaluate and compare the accuracy of different models in explaining the data. Models were compared using Bayes' factors, which impose a penalty for additional parameters. We interpreted our results with reference to the product of Bayes' factors (sum of deciBans) within and across treatments.

Our results provide very strong evidence that the immune response to *P. chabaudi* involves clearance of both parasitised and unparasitised RBCs. Both effects were evident during the initial peak of parasite density, implying that control of the peak is regulated by the immune response. Previous modelling studies have suggested that innate or early specific immune responses regulate the initial dynamics of parasite density and anaemia [8], [9], [21], [47]. Our study provides a statistically rigorous analysis in support of this hypothesis. Parasite-infected erythrocyte surface antigens (PIESA) may be an important immune target in both rodent and human malarias. In the case of *P. falciparum*, a longitudinal study of Kenyan children found that clinical malaria was caused by parasite isolates expressing PIESA variants that corresponded to gaps in the repertoire of antibodies carried by the children before they became ill [48].

We have shown that uRBC clearance by the immune system plays a key role in determining the infection dynamics of *P. chabaudi*. Experimental studies confirm that in the rodent malaria *P. berghei* [10], and also in *P. falciparum* which infects humans [11], the vast majority of anaemia is due to uRBC loss. Our results suggest that the level of anaemia following control of the initial peak (from about day 12), is mediated by the activity of the uRBC targeting response. This provides a mechanistic explanation for the variation in RBC dynamics between individual mice. If uRBC clearance decreases following control of the initial peak, then the increase in erythropoiesis that occurs from approximately day 10 allows the host to recover quickly from anaemia; in contrast, prolonged uRBC clearance is associated with a slow recovery from anaemia. Due to the lack of data to describe parasite density, the role of the pRBC targeting response during this later stage of infection is less certain. Our results suggest that the immune responses targeting pRBCs and uRBCs do not show a high degree of synchronisation. This implies they may be controlled by different effector mechanisms.

Our model does not account for the antigenic variation that occurs in *P. chabaudi* infections [49], [50]. Indeed, our model formulation only permits a single “switching-on” and “switching-off” of each immune component (merozoite, pRBC, uRBC), and therefore does not distinguish between non-specific (innate) versus specific antibody responses to antigenically distinct variants. We modelled the infection up until day 18, at which point antigenic variation may have a significant effect on the dynamics. In some mice, the data show a second drop in RBC density following the recovery from initial anaemia (Fig. 2). Although we only have data on parasite density up to day 14, this second anaemia is commensurate with a recrudescent parasite density. However, it is significant that our model is able to explain the observed dynamics so well without including antigenic variation (Fig. 5). Extending the model up to, for example, day 30 post-infection would require explicit modelling of immune responses to the different antigenic variants. Such modelling would probably need to include both short-lived (innate) and long-lasting antibody responses, and may also need to consider cross-reactivity. The cascade of sequentially dominant antigenic variants seen in *P. falciparum* infections has recently been explained as the result of short-lived cross-reactive immune responses directed against shared epitopes [36].

Our results are consistent with the observation that T-cell-deficient (“nude”) mice have impaired immune responses, and are unable to resolve malaria infections [2]. 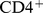 T cells play an important role during the early stages of malarial infection, by amplifying the phagocytic and cell-mediated antiparasite responses; later in the infection, they help B cells to produce antibody, and assist in regulating the innate response [51]–[53]. Immune-mediated clearance of uRBCs was necessary to explain the dynamics in all three phenotypes, but nude mice had the higher maximum clearance rate of uRBCs. The infection rate of RBCs with merozoites, as reflected in the parameter 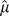, could also be higher in nude mice. As a simple proxy for the real biological system, these results indicate that nude mice are less able to limit the replication of the malaria parasite, and that their less specific immune response is associated with greater destruction of uninfected cells.

At the individual mouse level, there was no evidence for immune-mediated clearance of merozoites. However, the cumulative evidence over all mice suggests that it is required to explain our data. Our interpretation of this result is that merozoite clearance is weak during the first few weeks of infection, and that pRBC and uRBC clearance are the major determinants of the dynamics during this time. Previous models have shown that, for a given level of immune activity, merozoite clearance is less effective at controlling parasite density compared to an equivalent response that clears pRBCs [7], [8]. One explanation for this is that the duration of the merozoite infection phase (estimated to be on the order of minutes) is too short for the immune system to effectively target merozoites [7]. However, there is no *a priori* reason that one or several fast-acting, highly effective immune responses could not operate during this phase. There is considerable empirical evidence that merozoite surface protein one (MSP1) is a target of immune mechanisms in malaria infections [54]. The presence of high levels of naturally acquired IgG antibodies to merozoite surface protein two (MSP2) is also strongly associated with protection against clinical malaria [55]. Recent results suggest that this naturally acquired protection is not specific in relation to the major allelic dimorphisms of MSP2 [56].

We have shown that erythropoiesis upregulates during malarial infection, and that wildtype and reconstituted mice have higher upregulation than nude mice. This may reflect that the erythropoietic response only upregulates to the extent that the host is controlling the parasite. Recent theoretical results have shown that excessive upregulation of erythropoiesis facilitates the growth of the parasite, and may result in greater anaemia and a higher peak of parasite density [57]. We also investigated whether there is a time delay before the upregulation of erythropoiesis, and a lag in the feedback between RBC density and the level of erythropoiesis. The results for the AJ-infected wildtype mice suggest that upregulation of erythropoiesis occurs from day 10. Both reconstituted and nude mice show no evidence of a time delay. Our results also suggest a time lag of 2–3 days in the feedback between RBC density and erythropoiesis in AJ-infected reconstituted mice; however there is no evidence for a time lag in the other treatments. The reasons for this are unclear. One possibility is that our putative time lag is compensating for another process not included in the model. Only analysis of other data sets may reveal what this may be.

In summary, our results show that the immune system plays a key role in determining the RBC and parasite dynamics in malaria-infected mice. We have shown that immune-mediated clearance of both parasitised and unparasitised RBCs is necessary to explain the RBC and parasite dynamics. Previous models have examined the implications of RBC age structure for the infection dynamics [14], [22]. In particular, recent work by Mideo et al. (2008) suggests that *P. chabaudi* may preferentially infect mature RBCs (normocytes), but produce more merozoites in younger cells (reticulocytes) [14]. Future models may need to consider how RBC age structure and immune system dynamics can be combined to obtain a more complete picture of the asexual stage of malaria.

## Supporting Information

Figure S1 Parasite densities (cells/µl) measured by qPCR of two simultaneously taken samples used to estimate measurement error σ_p_.
(0.13 MB TIF)

Figure S2 Gelman-Rubin statistics for each parameter sorted by mouse (top panel) and by parameter (bottom panel).
(0.21 MB TIF)

Table S1 Bayes' factors for models π_1_ to π_8_ relative to model π_0_.
(0.01 MB PDF)

Table S2 Bayes' factors for models π_9_ to π_15_ relative to model π_0_.
(0.01 MB PDF)

Text S1 Mathematical appendix and statistical methods.
(0.07 MB PDF)
