# Supplementary figures and images for: Quantitative Analysis of Immune Response and Erythropoiesis during Rodent Malarial Infection
Source: PLoS Comput Biol. 2010 Sep 30;6(9):e1000946. doi: 10.1371/journal.pcbi.1000946 (PMC2947982; doi:10.1371/journal.pcbi.1000946)

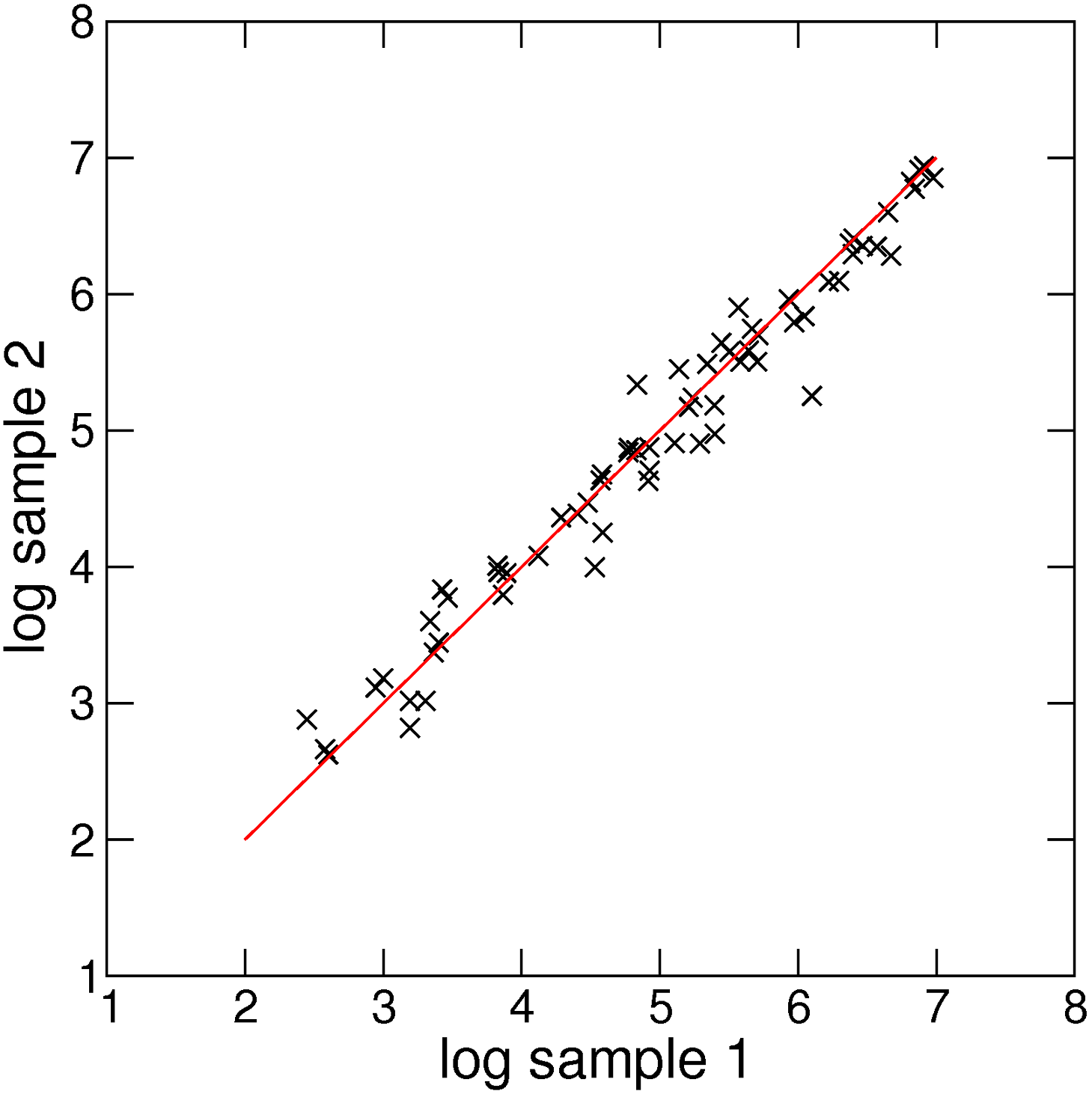

Supplement: Figure S1 — Parasite densities (cells/µl) measured by qPCR of two simultaneously taken samples used to estimate measurement error σp. (0.13 MB TIF) [file pcbi.1000946.s001.tif]

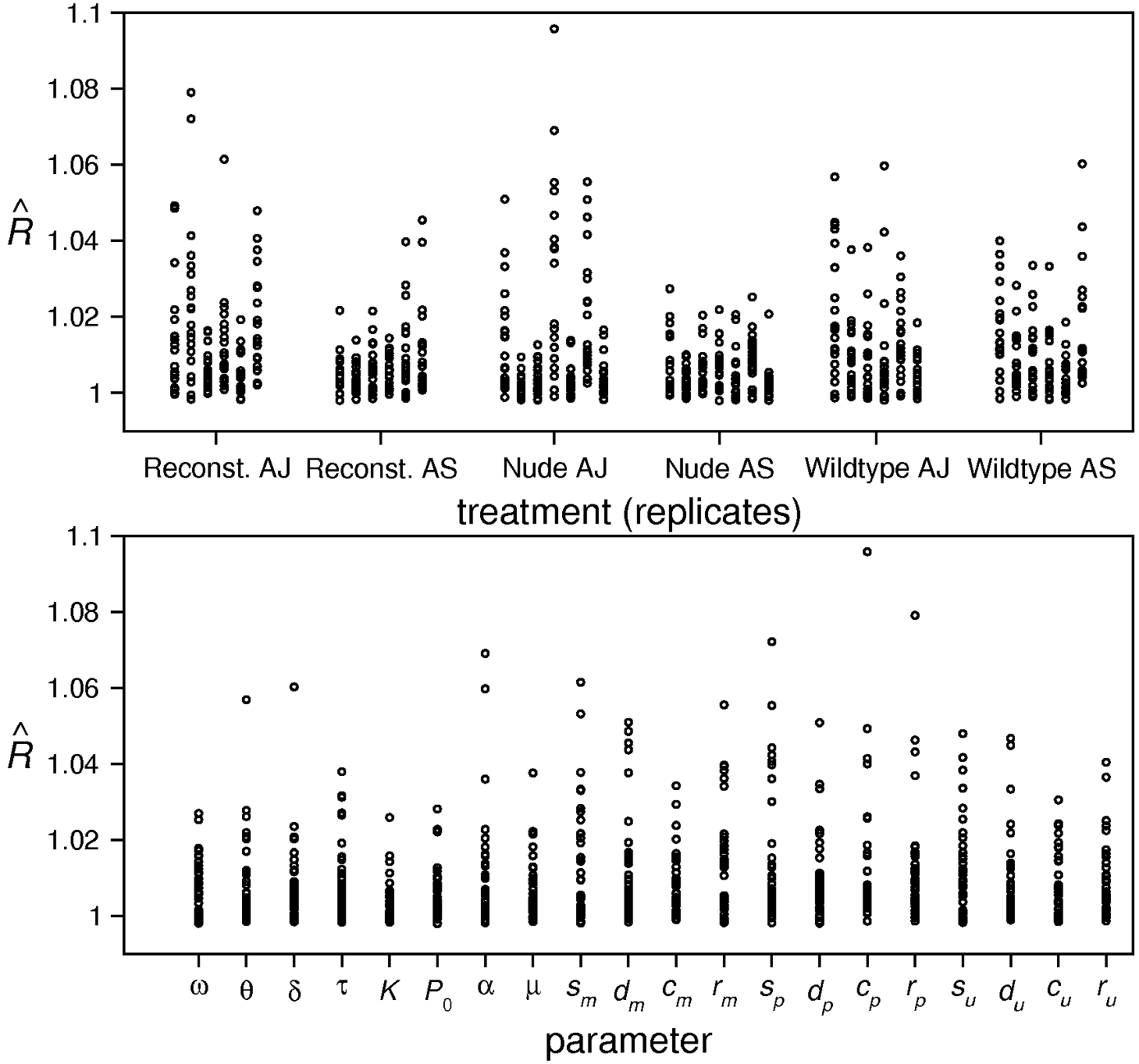

Supplement: Figure S2 — Gelman-Rubin statistics for each parameter sorted by mouse (top panel) and by parameter (bottom panel). (0.21 MB TIF) [file pcbi.1000946.s002.tif]
