# Supplementary material for: Quantitative Analysis of Immune Response and Erythropoiesis during Rodent Malarial Infection
Source: PLoS Comput Biol. 2010 Sep 30;6(9):e1000946. doi: 10.1371/journal.pcbi.1000946 (PMC2947982; doi:10.1371/journal.pcbi.1000946)
Supplement: Table S1 — Bayes' factors for models π1 to π8 relative to model π0. (0.01 MB PDF) [file pcbi.1000946.s003.pdf]

Table S1. Bayes' factors for models Π_1 to Π_8 relative to model Π_0.

Treatment	Mouse	Π_1	Π_2	Π_3	Π_4	Π_5	Π_6	Π_7	Π_8
Reconstituted AJ	1	0.9	5.3	16.0	-4.9	-0.7	6.6	3.2	2.1
	2	-3.8	5.2	11.8	0.8	0.8	2.8	3.9	2.4
	3	8.2	-2.1	29.1	-0.7	3.7	7.0	-0.5	-2.5
	4	0.4	7.5	16.3	5.0	1.8	7.6	6.9	-0.4
	5	4.3	0.0	18.1	-0.7	3.5	4.6	-0.4	-2.4
	6	0.7	8.8	3.0	3.5	1.7	7.1	4.1	0.2
Reconstituted AS	1	-1.5	6.7	-1.5	-0.7	-1.3	10.3	7.5	-0.4
	2	1.1	0.0	19.6	3.6	-0.3	6.8	1.8	-2.2
	3	6.9	-1.8	-2.4	-0.9	2.0	7.1	0.3	-2.0
	4	6.9	3.1	1.4	-0.6	0.5	5.5	1.8	1.8
	5	5.6	3.4	1.3	-2.7	-4.8	11.1	1.4	3.0
	6	1.8	0.2	3.4	-2.4	-5.3	5.6	0.6	-1.1
Nude AJ	1	1.6	18.0	5.0	13.1	0.4	21.2	8.8	-1.9
	2	-0.3	5.0	-1.4	3.3	-1.0	5.8	5.5	-1.8
	3	-1.1	4.6	-0.6	0.7	-0.4	4.4	4.5	0.1
	4	1.8	11.5	-1.9	1.7	-2.3	25.0	5.9	-0.2
	5	1.1	0.8	3.0	-1.4	-2.4	1.5	0.9	3.1
	6	1.1	8.9	2.0	-10.1	-7.8	12.0	7.5	0.2
	7	-0.7	4.7	0.8	1.1	-1.1	4.5	4.8	-0.1
Nude AS	1	1.8	5.2	2.2	0.1	-0.2	8.5	2.9	0.1
	2	0.6	2.0	6.8	-5.5	-6.4	7.2	1.1	6.4
	3	2.6	-2.2	15.1	0.8	-7.9	5.8	-2.0	4.4
	4	-0.6	0.8	0.1	-0.4	-2.4	9.7	1.2	0.0
	5	1.0	3.2	5.2	-3.9	-3.0	6.1	2.4	3.3
	6	-0.8	6.8	1.6	-1.0	0.5	7.7	6.1	0.6
	7	1.3	0.3	5.2	-0.6	-4.0	6.6	0.8	3.6
Wildtype AJ	1	5.7	20.7	50.3	8.1	11.5	5.2	3.0	36.8
	2	10.0	11.7	65.6	0.7	-4.0	16.9	8.5	0.1
	3	-0.5	6.9	77.9	4.7	16.1	2.7	7.2	32.5
	4	2.5	15.3	34.2	7.8	12.1	3.0	4.6	26.4
	5	-0.5	5.9	55.7	-3.2	8.9	5.7	5.1	28.2
	6	-1.5	13.3	7.5	-2.0	2.2	5.9	10.7	-0.1
Wildtype AS	1	5.4	26.3	48.3	13.3	8.8	8.3	6.3	52.8
	2	-2.2	10.5	14.9	-2.4	3.5	1.2	6.4	8.5
	3	3.2	7.1	57.1	6.0	18.7	-2.2	4.2	24.1
	4	0.4	14.7	49.6	-3.9	11.3	6.4	11.0	15.6
	5	11.8	-2.1	127.3	5.2	7.9	2.3	-1.6	48.5
	6	-0.4	10.9	48.7	-2.2	10.8	-0.2	8.1	21.3
