# Supplementary material for: Quantitative Analysis of Immune Response and Erythropoiesis during Rodent Malarial Infection
Source: PLoS Comput Biol. 2010 Sep 30;6(9):e1000946. doi: 10.1371/journal.pcbi.1000946 (PMC2947982; doi:10.1371/journal.pcbi.1000946)
Supplement: Table S2 — Bayes' factors for models π9 to π15 relative to model π0. (0.01 MB PDF) [file pcbi.1000946.s004.pdf]

Table S2. Bayes' factors for models Π_9 to Π_{15} relative to model Π_0.

Treatment	Mouse	Π_9	Π_{10}	Π_{11}	Π_{12}	Π_{13}	Π_{14}	Π_{15}
Reconstituted AJ	1	-1.9	674.0	-0.3	3.7	1.4	3.1	0.4
	2	-3.6	706.4	-2.5	-7.9	-11.1	-3.9	1.0
	3	5.8	482.7	-1.3	17.2	26.8	11.0	-0.5
	4	0.2	426.8	-0.5	3.3	2.7	9.5	0.6
	5	5.4	395.9	-0.2	9.2	32.1	13.5	-0.9
	6	-2.7	664.7	-1.2	-7.3	-8.3	4.6	0.8
Reconstituted AS	1	0.0	204.6	0.5	0.3	2.9	8.1	0.3
	2	3.1	94.8	-0.1	-0.3	3.7	7.4	-1.5
	3	7.9	187.8	0.2	-5.6	-5.4	12.6	-1.1
	4	5.6	80.9	0.1	-3.0	-2.5	5.4	-0.3
	5	3.5	358.9	-0.2	3.2	3.1	7.1	-0.7
	6	1.4	566.3	-1.9	5.9	6.4	6.2	-3.2
Nude AJ	1	1.4	222.1	-1.8	-2.6	-4.7	2.8	-0.6
	2	-0.7	0.8	0.4	0.0	0.2	5.4	-0.1
	3	-0.4	3.4	-1.0	-3.6	-5.4	8.3	-0.1
	4	1.3	341.4	-0.9	-0.9	-1.0	5.3	-1.0
	5	1.1	2.2	-0.4	-1.8	-1.3	7.3	-0.1
	6	-0.1	263.9	1.2	-2.6	-4.3	3.8	2.1
	7	0.0	3.6	0.4	-3.2	-4.8	11.7	-0.5
Nude AS	1	0.2	2.4	-0.2	-6.7	-7.5	3.8	-0.5
	2	1.0	21.7	-1.6	-3.4	-4.6	2.8	0.2
	3	3.9	42.1	-2.0	1.4	3.6	6.0	-2.0
	4	1.2	9.5	-1.3	-0.9	-1.6	8.5	-1.6
	5	1.0	6.5	-0.7	-6.3	-7.9	5.4	0.1
	6	0.0	-1.6	-0.4	-3.5	-4.9	5.9	0.3
	7	2.3	11.8	-0.6	-2.1	-3.7	7.2	-1.8
Wildtype AJ	1	-1.9	336.8	2.1	-8.8	-8.6	2.0	0.9
	2	-0.7	301.7	-2.0	-2.2	-3.2	10.3	0.7
	3	-0.7	344.7	2.8	8.8	28.4	6.0	0.3
	4	-1.2	371.2	-0.5	-9.0	-9.6	2.0	1.2
	5	0.2	280.7	1.2	8.4	21.2	3.9	0.7
	6	-0.5	488.2	-1.8	-2.1	-3.3	7.7	2.3
Wildtype AS	1	-1.6	230.1	-0.9	-8.2	-9.3	0.4	0.2
	2	-1.2	204.8	-1.3	-4.1	-1.7	5.2	-1.4
	3	0.3	177.2	3.6	-9.2	-2.2	5.7	0.6
	4	0.2	269.4	1.3	1.3	8.5	6.4	1.1
	5	8.8	169.6	14.3	6.7	42.7	0.3	-0.4
	6	-1.4	178.8	4.7	-7.9	5.6	3.0	-1.7
