# Supplementary material for: Quantitative Analysis of Immune Response and Erythropoiesis during Rodent Malarial Infection
Source: PLoS Comput Biol. 2010 Sep 30;6(9):e1000946. doi: 10.1371/journal.pcbi.1000946 (PMC2947982; doi:10.1371/journal.pcbi.1000946)
Supplement: Text S1 — Mathematical appendix and statistical methods. (0.07 MB PDF) [file pcbi.1000946.s005.pdf]

Text S1: Mathematical appendix and statistical methods

Derivation of RBC turnover equations

Here we determine the solution to the system of equations (11)-(15) describing the RBC turnover phase. Consider first the case where $i > \delta$. Equation (11) describing the change in uRBC density can be written as

$$\frac{dU(s)}{ds} + (d + I_{u,i})U(s) = \theta(K - N_{i-\tau}) + dK \tag{S.1}$$

This is solved by introducing the integrating factor, $\exp((d + I_{u,i})s)$, and using the identity

$$\frac{d[U(s)\exp((d + I_{u,i})s)]}{ds} = \left(\frac{dU(s)}{ds} + (d + I_{u,i})U(s)\right)\exp((d + I_{u,i})s) \tag{S.2}$$

Combining (S.1) and (S.2) gives

$$U(s)\exp((d + I_{u,i})s) = (\theta(K - N_{i-\tau}) + dK)\int \exp((d + I_{u,i})s)ds \tag{S.3}$$

Integration of (S.3) gives the solution

$$U(s) = \frac{\theta(K - N_{i-\tau}) + dK}{d + I_{u,i}}(1 - \exp(-(d + I_{u,i})s)) + U(0)\exp(-(d + I_{u,i})s) \tag{S.4}$$

If $i \leq \delta$, the term $\theta(K - N_{i-\tau})$ in (S.4) is omitted. Equations (12)-(15) are solved using similar methods, giving the following solutions:

$$P_1(s) = P_1(0)\exp(-(d + I_{p,i})s) \tag{S.5}$$
$$P_2(s) = P_2(0)\exp(-(d + d_m + I_{p,i})s) \tag{S.6}$$
$$p_1(s) = p_1(0)\exp(-(d + I_{p,i})s) \tag{S.7}$$
$$p_2(s) = p_2(0)\exp(-(d + d_m + I_{p,i})s) \tag{S.8}$$

Measurement errors in RBC and parasite densities

We fit to the RBC densities using the model:

$$N_{\text{data}} = N_{\text{model}} + \epsilon_N, \qquad \text{where } \epsilon_N \sim N(0, \sigma_N^2) \tag{S.9}$$

We fit to the measured parasite densities using the model:

$$\log p_{\text{data}} = \log p_{\text{model}} + \epsilon_p, \qquad \text{where } \epsilon_p \sim N(0, \sigma_p^2) \tag{S.10}$$

We estimated the variances in the measurement errors as follows. RBC densities were measured using flow cytometry [1]. We have previously estimated the standard deviation of the error, σ_N, of this method to be 0.61×10^6 (95% CI: $0.44 \times 10^6,\ 0.84 \times 10^6$) cells/$\mu$l [2]. Parasite densities were measured using quantitative PCR [1]. In another experiment using the same protocol as here (A. Bell, personal communication) two blood samples were simultaneously taken from infected mice at various time points (Figure S1). Assuming that parasite density is log-normally distributed with standard deviation σ_p, the difference in two simultaneously taken samples is log-normally distributed with mean 0 and standard deviation $\sqrt{2}\sigma_p$. We therefore estimate σ_p to be 0.15. The errors in the samples are consistent with normality (Andersen-Darling statistic: $A^2 = 1.08, p > 0.1$). Taking a prior on σ_N as $\text{N}(0.6 \times 10^6, 0.1)$ and a prior on σ_p as N(0.15, 0.1) we re-estimated their values by fitting to the data in this paper. The new estimates are $\sigma_N = 5.0 \times 10^5 \pm 1.3 \times 10^5$ cells/μl and $\sigma_p = 0.130 \pm 0.002$.

Priors

Burst size, ω, is estimated to be 6-8 merozoites per pRBC [3]. We thus take its prior as $\mathrm{N_T}(7, 0.5)$. The rate of upregulation of erythropoiesis, θ, is estimated to be about 0.2 day^{-1} in [4] (this previous study uses different parasite strains) and 0.4 day^{-1} in [5]. We thus take its prior to be $\mathrm{N_T}(0.28, 0.1)$. The delay prior to upregulation, δ, is estimated to be about 2.5 days (95% CI: 0-5 days) after anaemia is chemically induced in mice [2]. In malaria infections, anaemia begins from about day 5. We thus take a wide prior on δ of U(0, 12). The erythropoietic time lag, τ, is estimated to be between 1-3 days [5, 6] but with significant variation between mice. We take a prior of U(0, 6) to cover this range. The normal RBC density, K, can be approximated from the first few days of the experiment using all mice. The mean density is 1.06×10^7cells/μl and the standard deviation is 0.10×10^7cells/μl. We thus take a prior of $\mathrm{N_T}(1.06 \times 10^7, 0.1 \times 10^7)$. Each mouse was infected with 10^5 parasites. Mice have roughly 3 ml of blood, therefore the initial parasite density was about 33 parasites/μl, or approximately 1.5 on a log-scale. Given uncertainty about how many parasites enter the circulation from the peritonea into which they are innoculated, we take a broad prior on $\log(P_0)$ of $\mathrm{N_T}(1.5, 0.5)$. The death rate of multiply-parasitised RBCs, d_2, is unknown. We therefore assume a prior on α of U(0, 1). The natural loss rate of merozoites μ is unknown. However, $\hat{\mu}$ must be of the same order of magnitude as the RBC density, N, to have an effect on the dynamics (see Equation 6 in the main text). Given that the maximum of N is K, which is of order 10^7, we assume a broad prior on $\hat{\mu}$ of $\mathrm{U}(0, 2 \times 10^7)$.

There are no estimates in the literature for the immune response parameters, and we therefore assumed broad priors. For merozoite clearance to have an effect on the dynamics, c_m must be of the order of K (see Equation 6 in the main text). We therefore take a prior of $\mathrm{N_T}(10^7, 10^7)$. For pRBC and uRBC clearance to have an effect on the dynamics, c_p and c_u must be on the order of λ, the average number of parasites per RBC (see Equations 16 and 17 in the main text). Near to the peak of parasite density, this is of the order 1. We therefore assume priors on c_p and c_m of $\mathrm{N_T}(1, 1)$. The start times, rise times and durations of the immune responses could be anywhere between the start and end of the experiment. We therefore take their priors as U(0, 19).

Model fitting and parameter estimation

For each mouse, the log-likelihood of a model with parameter vector Φ given the data is, up to a constant of proportionality

$$\ln L(\Phi) \propto -\frac{1}{2}\left(\sum_{j=1}^{T_N}\left(\frac{N_{\mathrm{data},j} - N_{\mathrm{model},j}(\Phi)}{\sigma_N}\right)^2 + \sum_{j=1}^{T_p}\left(\frac{\log p_{\mathrm{data},j} - \log p_{\mathrm{model},j}(\Phi)}{\sigma_p}\right)^2\right) \tag{S.16}$$

Here T_N is the number of data points for RBC density, $N_{\mathrm{data},j}$ is the measured RBC density at time point j, and $N_{\mathrm{model},j}(\Phi)$ is the model solution of RBC density at time point j. Similarly, T_p is the number of data points for parasite density, $p_{\mathrm{data},j}$ is the measured parasite density at time point j, and $p_{\mathrm{model},j}(\Phi)$ is the model solution of parasite density at time point j.

Evolutionary Monte Carlo method

We use the Evolutionary Monte Carlo (EMC) method (without crossover) to sample posterior densities [7,8]. Two significant advantages of this method are its excellent characterisation of multi-modal posterior densities and reliable estimates of marginalised likelihoods of the data, and thus Bayes' factors [9, 10].

In the EMC method there are B tempered densities with inverse temperatures $t_1, \ldots, t_B$, which form a ladder with the ordering $t_1 > \ldots > t_B$. A challenge of EMC is to find the optimal form of this ladder and the number of densities B such that the minimum state-exchange rate between neighbouring densities remains high (above 50% [7]). Below this value, marginalised likelihoods become inaccurate and

imprecise. For this data set we have found that $B = 40$ and a temperature ladder of the form $t_i = t_2^{(i-1)^A}$ for $i = 3, \ldots, B-2$, where $A = \ln(\ln(t_{B-1})/\ln(t_2))/\ln(B-2)$, $t_1 = 1$, $t_2 = 0.9$, $t_{B-1} = 10^{-4}$ and $t_B = 0$ gives satisfactory results.

We block-update all model parameters using the Metropolis algorithm. The transition distributions of the algorithm are multivariate normal with mean 0 and covariance matrices S_i, for $i = 1, \ldots, B$. The covariance matrices are estimated from the empirical covariance matrices of the Markov chains during an adaptive McMC phase [11]. The adaptive algorithm works as follows:

1: Set off-diagonal elements of S_i to 0; set diagonal elements to 1/500th the scale of the prior densities (the densities at the initial parameter guesses).

2: Every 100 iterations calculate the empirical S_i matrices and their Cholesky decompositions. Tune their scales (by factors of 0.5 and 2) to keep the chains' acceptance rates above 20% and below 40% respectively [12, 13]. Exit when 500 unique samples have been collected and the acceptance rates have stabilised between 20% and 40%, i.e., have not moved out of this range over 200 iterations.

Four Markov chains are run simultaneously and convergence to the posterior is assessed using the Gelman-Rubin statistic on each parameter [14]. We use 2,000,000 iterations of a non-adaptive McMC phase; the first 50% are discarded as burn-in and the the rest thinned to 1 in 500, giving 2,000 samples on which to base inferences.

Models are assessed by three methods: calculation of residual deviance, visual inspection of fits to data, and plotting overlaid standardised residuals. Visual inspection of fits highlights gross inadequacies within and across replicates, but is not particularly sensitive. Standardised residuals provide a more sensitive assessment of fit. Poor fits exhibit outliers and serial correlation. By overlaying plots, systematic biases at single time points are also revealed (see below).

The EMC algorithm, the models and the various diagnostics and statistics are implemented in the C programming language on the Linux operating system, and complied using the Intel® C/C++ compiler. Diagnostic visualisation is coded in the Python programming language coupled to the GRACE graphics plotting package. We use the OpenMP Application Program Interface.

Assessment of model adequacy

We assess a model's ability to predict the data using marginal standardised residuals. For data point j the marginal standardised residual is:

$$r_j = \frac{1}{\sigma_x} \int_{\Phi} (x_{\text{data},j} - x_{\text{model},j}(\Phi)) d\Phi \tag{S.17}$$

where x is either N for RBC density or p for parasite density.

For the true model, standardised residuals are approximately normally distributed with mean 0 and standard deviation 1. Although they are not strictly independent and identically distributed, we can treat them as such for the purposes of model assessment, which by its nature is subjective. Poor fits to data therefore exhibit outliers (larger than 3 standard deviations from 0) and serially correlated residuals.

We can also look for systematic biases in fits across mice because their RBC and parasite dynamics are sufficiently similar over time. We do this by overlaying the standardised residual plots for each mouse and plotting the mean of the standardised residuals at each time point. For the true model the mean of n standardised residuals is approximately normally distributed, with mean 0 and standard deviation $1/\sqrt{n}$. We can therefore examine, for each time point, if the mean of the standardised residuals of n mice lies within the 95% prediction interval $(-1.96/\sqrt{n},\ 1.96/\sqrt{n})$. The interval is Bonferonni corrected to adjust for multiple time points. Mean standardised residuals that lie above (below) the interval suggest systematic underestimation (overestimation) of the data across all mice.

Model comparison

Model fits are compared using Bayes' factors, which impose a penalty for additional parameters [15]. Each model, Π_j, is compared to the baseline, Π_0. Bayes' factors are calculated by evaluating the marginal likelihood of each model, Pr(data|model). The Bayes' factor for model Π_0 over model Π_j is:

$$\text{(Bayes' factor)}_j = \frac{\Pr(\text{data}|\Pi_0)}{\Pr(\text{data}|\Pi_\text{j})} \tag{S.18}$$

This is more conveniently expressed in deciBans (tenths of a power of 10):

$$(\text{dB})_j = 10(\log \Pr(\text{data}|\Pi_0) - \log \Pr(\text{data}|\Pi_\text{j})) \tag{S.19}$$

Positive deciBan values imply that Π_0 explains the data better than Π_j. Negative deciBan values imply that Π_j explains the data better than Π_0 [16]. We adopt the scale of interpretation given by Jeffreys [15], which is reproduced in the main text.

Because we use EMC to estimate marginal likelihoods, there is inevitably sampling error in their estimates. This feeds through into estimates of deciBans. We estimated the error in deciBans by running 100 simulations of the models and calculating the standard deviation of the marginal likelihoods for each mouse.

Assessment of Markov chain convergence

Figure S2 shows the Gelman-Rubin statistics [14] for each parameter sorted by mouse (top panel) and by parameter (bottom panel). Almost all values are close to 1, suggesting good convergence of the four Markov chains to the posterior.

References

1. Råberg L, de Roode JC, Bell AS, Stamou P, Gray D, et al. (2006) The role of immune-mediated apparent competition in genetically diverse malaria infections. Am Nat 168: 41–53.

2. Savill NJ, Chadwick W, Reece SE (2009) Quantitative analysis of mechanisms that govern red blood cell age structure and dynamics during anaemia. PLoS Comput Biol 5: e1000416.

3. Carter R, Diggs CL (1977) Plasmodia of rodents. In: Kreier JM, editor, Parasitic protozoa, New York: Academic Press, volume 3. pp. 359–461.

4. Haydon DT, Matthews L, Timms R, Colegrave N (2003) Top-down or bottom-up regulation of intra-host blood-stage malaria: do malaria parasites most resemble the dynamics of prey or predator? Proc Roy Soc Lond B 270: 289–298.

5. Mideo N, Barclay V, Chan BHK, Savill N, Read A, et al. (2008) Understanding and predicting strain-specific patterns of pathogenesis in malaria, *Plasmodium chabaudi*. Am Nat 172: E214–E238.

6. Chang KH, Tam M, Stevenson MM (2004) Modulation of the course and outcome of blood-stage malaria by erythropoietin-induced reticulocytosis. J Infect Dis 189: 735–743.

7. Liang F, Wong WH (2000) Evolutionary Monte Carlo sampling: Applications to C_p model sampling and change-point problem. Stat Sinica 10: 317–342.

8. Liang F, Wong WH (2001) Real-parameter Evolutionary Monte Carlo with applications to Bayesian mixture models. J Am Stat Assoc 96: 653–666.

9. Lartillot N, Philippe H (2006) Computing Bayes' factors using thermodynamic integration. Syst Biol 55: 195–207.

10. Friel N, Pettitt AN (2008) Marginal likelihood estimation via power posteriors. J Roy Stat Soc B 70: 589–607.

11. Haario H, Saksman E, Tamminen J (2001) An adaptive Metropolis algorithm. Bernoulli 7: 223–242.

12. Gelman A, Carlin JB, Stern HS, Rubin DB (2004) Bayesian Data Analysis. London: Chapman and Hall.

13. Andrieu C, Thoms J (2008) A tutorial on adaptive MCMC. Stat Comput 18: 343–373.

14. Gelman A, Rubin DB (1992) Inference from iterative simulation using multiple sequences. Stat Sci 7: 457–511.

15. Jeffreys H (1961) The Theory of Probability. University of Oxford: Oxford University Press, third edition.

16. MacKay DJC (1992) Bayesian interpolation. Neural Comput 4: 415–447.
